# Psychological care in acute and emergency medicine: a scoping review of support interventions by healthcare professionals

**DOI:** 10.1186/s12873-026-01494-y

**Published:** 2026-02-18

**Authors:** Johanna Rutetzki, Jessica Breuing, Käthe Goossen, Franka Lucius, Thomas Ostermann, Katharina Fetz

**Affiliations:** 1 Institute for Research in Operative Medicine (IFOM), Witten/Herdecke University, Cologne, Germany; 2 Department of Psychology, Chair of Research Methodology and Statistics in Psychology, Witten/Herdecke University, Witten, Germany; 3 Medical Faculty, Heinrich-Heine University, University Hospital, Düsseldorf, Germany

**Keywords:** Communication, Emergency medicine, Emergency treatment, Patient care, Psychological first aid, Psychological intervention, Psychological trauma

## Abstract

**Background:**

Medical emergency situations can be highly distressing for patients and potentially lead to the development of psychological disorders. While psychological care and interventions are of obvious necessity to assist patients when coping with emergency situations, research regarding appropriate psychological care of prehospital and clinical emergency patients is limited. This scoping review provides an overview of the current state of research in emergency medicine with particular focus on communication strategies as well as psychological interventions. The aim is to present an overview of the psychological support recommended for or available to emergency patients.

**Methods:**

PubMed and PsycInfo were searched for eligible articles on psychological interventions or communication styles that assist acute and emergency adult patients in coping with the critical incident. The search started in June 2023 and was repeated in June 2024.

**Results:**

The literature search resulted in 1080 articles. Ultimately, 26 articles were eligible for inclusion. The majority of articles focused on major and general trauma patients. Study settings involved emergency rooms, emergency medical services, burn units, intensive care units and other acute medical settings, such as maternity wards. While research results are heterogeneous due to a wide variety of interventions, there is evidence that patients require (psycho-)education and information regarding diagnosis and treatment to reduce psychological distress and maintain or regain mental health.

**Conclusions:**

This scoping review shows that the existing interventions and recommendations are too heterogeneous to recommend a universal approach for healthcare professionals. Nevertheless, (psycho-)education and informing patients has been demonstrated to be an effective means of reducing patients’ psychological distress and maintaining mental health. Further research is required to identify the most effective methods for assisting patients.

**Registration number:**

This scoping review was registered with the Open Science Framework on 29 May 2024 (DOI: 10.17605/OSF.IO/GJ4EX). Clinical trial number: not applicable.

**Supplementary information:**

The online version contains supplementary material available at 10.1186/s12873-026-01494-y.

## Background

### Rationale

Emergency medicine traditionally focuses on physical survival of patients and has significantly developed over the years [1]. While physical treatment has improved, another important aspect of patient care has been overlooked: the psychological effect of a medical emergency and its consequences for the mental health of patients [2, 3].

It is well documented that medical emergency situations such as being in an ambulance or emergency room can be very stressful for patients [4, 5]. Stress as defined by Lazarus and Folkman (1984) is a psychological state that arises when an individual is confronted with a challenging situation and is unable to effectively cope with it. According to this model, the degree of stress experienced by patients in response to a medical emergency depends upon their interpretation of the situation and the resources available to them to cope with it. In addition, the capacity to cope with stress can determine the extent of psychological difficulties experienced [6]. Acute medical emergencies, as potential traumatic events, can induce feelings of helplessness, fear or fright, and can lead to psychological stress or psychological disorders [7]. A study conducted by Faessler et al. (2016) found that some forms of psychological distress were present in approximately 38% of patients 30 days after being discharged from the emergency room [8]. A systematic review revealed that up to 47% of medical emergency patients exhibited psychological distress [9]. Memory loss is a common co-occurrence in critically ill patients and is positively associated with the level of post-traumatic stress disorder (PTSD) [10, 11]. Following orthopaedic trauma, 51% of patients met the criteria for PTSD [12]. Perruche et al. (2011) found that symptoms of anxiety occurred in 47% of emergency patients, and 23% demonstrated indications of depression [13]. Mental disorders such as PTSD and depression are associated with significant impairments in functional outcomes, including the inability to return to work [14] and more postoperative complications, such as acute renal failure, sepsis or deep venous thrombosis [15].

Acute medical emergencies can be defined as a traumatic event following the Diagnostic and Statistical Manual of Mental Disorders (DSM-5) first criteria of the PTSD criteria: Confrontation with actual or imminent death, serious injury or sexual violence [16]. In light of the high prevalence of psychological distress in the aftermath of medical emergencies, this study conceptualises every medical emergency as the potential occurrence of a traumatic event.

Although physicians are able to provide effective physical care to emergency patients, their abilities to diagnose and address underlying psychological conditions such as anxiety and depression are limited [13]. The provision of psychological support for critically ill patients is a matter of great importance to healthcare professionals [17]. Furthermore, current clinical guidelines recommend screening and psychological care for patients who have experienced traumatic events [18]. Despite the evident necessity of psychological care and interventions to help process medical emergency situations, there is limited research investigating evidence-based, appropriate psychological handling of prehospital and clinical emergency patients [3, 19].

A number of systematic reviews has been conducted on the subject of psychological interventions for acute and emergency patients. However, these reviews focused on specific disorders, such as acute and posttraumatic stress disorder, and specific patient populations [20–22]. The scope of this review is not limited to a specific injury or disease, nor to a single specific setting. The emergency department is a multifaceted department comprised of various sub-departments, including, but not limited to the trauma department, the burn department, the internal medicine, the intensive care, the emergency medical service, the labour and delivery unit and other emergency-related departments [23]. Common in all these specialities is the regular occurrence of medical emergencies.

Healthcare professionals working with emergency patients have various professions. The first contact to emergency healthcare might be the Emergency Medical Services (EMS). Depending on the emergency and the country, the professions are such as Emergency Medical Technicians (EMT) and emergency physicians [24, 25]. In the emergency department are mostly nurses and physicians of various specialties working [26–28]. Some patients would need specialized emergency healthcare, such as healthcare professionals specialising in intensive care [29], burn care [30, 31], or obstetrical care [32, 33]. Within these specialized departments, additional healthcare professionals, including midwives [34] and psychologists [35, 36], are actively involved in patient care.

Prehospital emergency medicine is defined as any professional intervention for acute life-threatening conditions performed in an out-patient setting or prior to admission to a hospital, e.g. during ambulance transport [37]. Clinical emergency medicine is defined as occurring in the clinical setting, such as the emergency room or other related units [38].

When patients first enter the healthcare system, psychological care is particularly challenging due to limited knowledge of their individual needs and preferences. In this context, patients must place trust in healthcare professionals who provide support through communication strategies or interventions despite this lack of prior information.

Additionally, psychological first aid is defined as occurring within the first hours and days conducted by professionals not specialized in emergency psychology, such as the emergency medical services [39]. An emergency medical patient might encounter a variety of professions, the emergency medical care can be described as multidisciplinary. Psychological care is not confined to a single profession, all professions that interact with emergency patients can utilize supporting communication strategies and interventions. Early support for patients is critical because it has the potential to limit the psychological consequences of medical emergencies.

### Objectives

The objective of this review was to identify effective communication strategies and psychological interventions, such as verbal, nonverbal and paraverbal communication strategies, explanations about medical treatments, injuries and accident mechanisms that are easily understood, as well as methods to assist with memory loss, and further specific psychological approaches. These interventions should assist patients in processing their experience of their medical emergency and reducing psychological distress, as well as maintaining or regaining mental health.

Therefore, the research question of this scoping review was as follows: What communication strategies or psychological interventions can healthcare professionals use during the initial contact phase within an emergency setting to provide psychological support to emergency patients?

## Methods

### Protocol and registration

This scoping review was registered with the Open Science Framework on 29 May 2024 (DOI: 10.17605/OSF.IO/GJ4EX). The Framework of Arksey & O’Malley (2005) was used to guide the review process [40].

### Eligibility criteria

The eligibility criteria were categorized using the Population, Concept, Context (PCC) mnemonic [41], as illustrated in Table 1.

**Table 1:** Population, Concept, Context (PCC)

POPULATION	Emergency patients	Patients with serious/life-threatening injuries/diseases
	Treated for psychological aspects of somatic emergencies	Treated in an acute/subacute phase (within 72 hours after initial contact to medicine) by any healthcare professionals, e.g. nurses, physicians, psychologists, social workers, paramedics
CONCEPT	Psychological Intervention	Psychoeducation, calming techniques…
	Communication style	E.g. informative, calm voice, reassuring…
CONTEXT	Acute and emergency medical contexts, clinical and prehospital settings	Emergency department
		Emergency medical services
		Burn department
		Trauma department
		Intensive care unit
		Other acute and emergency context

We only included articles about acute and emergency settings, such as emergency rooms or EMS to focus on patients experiencing psychological stress because of their acute health situation. The aim of this review was to identify how healthcare professionals can assist in acute emergency situations, rather than in the aftermath. Therefore, articles were considered eligible if they presented a communication strategy or psychological intervention that is intended to maintain mental health or support patients in crisis. The strategy or intervention must be applicable within a period of 72 hours following the initial contact with the health system rather than being long-term interventions that are mostly conducted outside of emergency situations. This is in line with the objective of identifying strategies and interventions that healthcare professionals can utilize to assist patients within the initial contact phase to emergency healthcare. Furthermore, as psychological first aid is conducted by professionals not specialized in emergency psychology [18, 39, 42], healthcare professionals, such as nurses, physicians, or paramedics must have carried out the communication strategy or psychological intervention.

Given the differing approaches to paediatric and adult patient care and family support, only studies focusing on adult patients ( > 18 years) were included in this review. In order to gain a comprehensive understanding of the published research, no limitations were imposed on the publication date nor the source of articles. Due to the constraints of temporal resources, we only screened articles in English or German.

Articles were excluded if they included patients below the age of 18, lacked a recommendation for healthcare professionals, or if they did not treat emergency patients with a focus on psychological management. A list of the articles that were excluded from the full-text reading is provided in the supplementary material 1.

### Information sources and search

Following the “Preferred Items for Systematic Reviews and Meta-Analysis extension for Scoping Reviews” (PRISMA-ScR) checklist [43] (see the supplementary material 3), a search was conducted on PubMed in June 2023 and PsycInfo in April 2024 using the search terms (“emergency patient*” OR “trauma patient*”) AND (“communication” OR “psycholog*” OR “psychological first aid”). The search term was not adapted for PsycInfo database. The search on PubMed was repeated in June 2024.

The research team (JR, KF, FL, KG, JB) designed an initial search strategy, then complemented it by terms obtained using the Systematic Review Accelerator (SRA) word frequency analyser [44]. The recommended items from the SRA did not improve the search term. Therefore, they were not incorporated into the final version. The PubMed forward citation search was concluded in June 2024. In April 2024, we initiated a search on PsycInfo using the same search term. A reference list checking and forward citation search was conducted using the selected eligible articles. The forward citation search was concluded in February 2025.

### Selection of sources of evidence

**Fig. 1:**
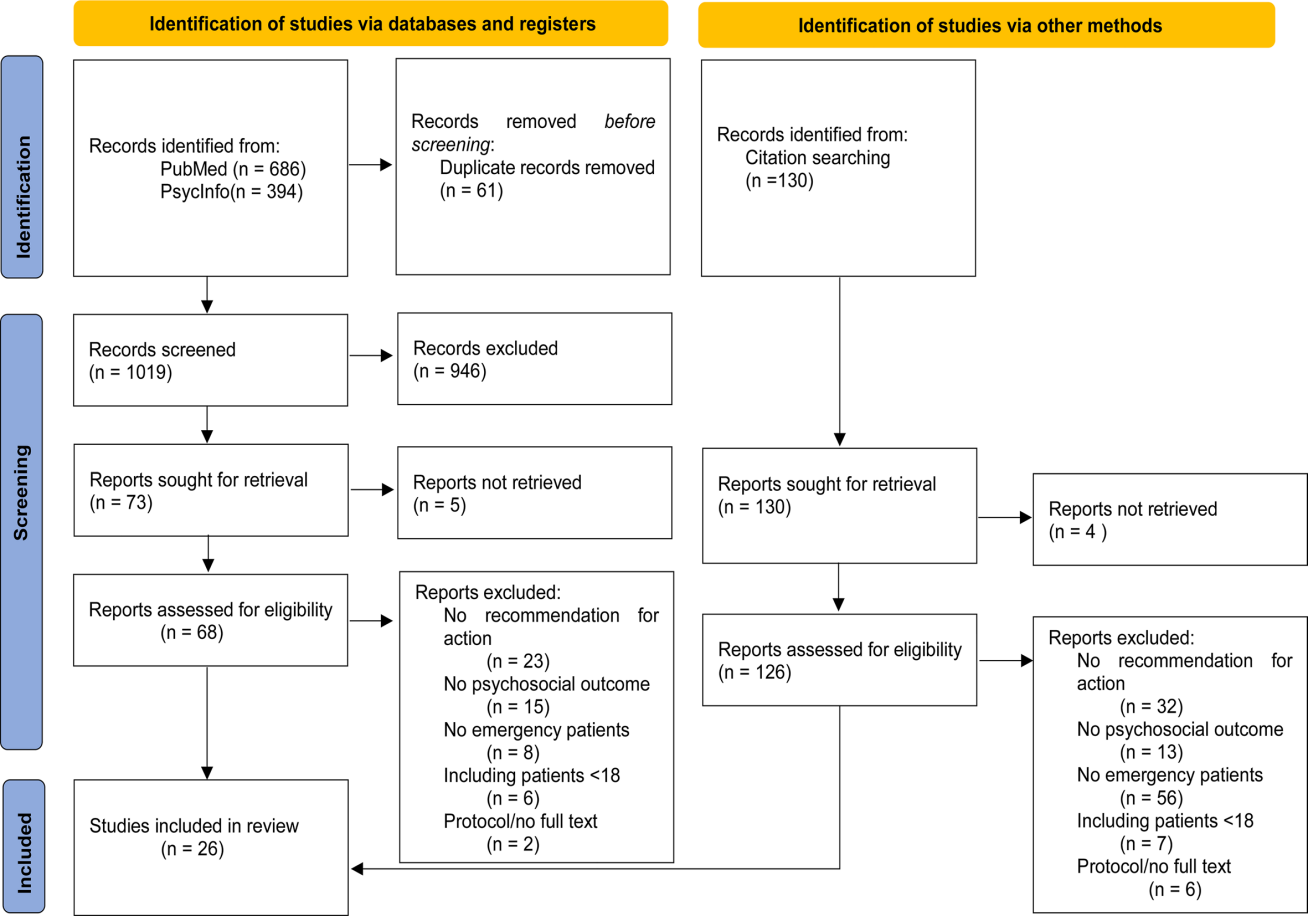
PRISMA flowchart

Two researchers (JR, FL) selected the articles independently, following the inclusion and exclusion criteria. Firstly, each researcher independently scanned the titles and abstracts of the search. The full texts of potentially relevant articles were then retrieved and independently scanned for eligibility by the two researchers. Any discrepancies were resolved through discussion and consensus between the two researchers (JR, FL). The reasons for the exclusion of full texts were documented in Fig. 1, the PRISMA flowchart [45], which can be seen in the results.

### Data charting process

In order to ensure the screening and traceability of each article, all sources were exported into a Microsoft Excel file, in which the researchers (JR, FL) marked the included, excluded and discussed articles.

### Data items

One researcher (JR) compiled the included articles into tables according to their setting, which are provided in the supplementary material 2. Another researcher (KF) crosschecked the extracted data. Each article was described with the following information, if the information was reported within the article:

- the intervention that was tested, described or wished for
- the population of the article
- the number of patients
- the provider of the intervention
- the survey period
- the publication year
- the country of study
- the study design
- the main outcome/key message

### Critical appraisal of individual sources of evidence

Two researchers (JR, AT) independently assessed the risk of bias of included RCTs using the Risk of Bias 2 (RoB 2) tool [46]. This tool includes assessment of risk of bias arising from the randomization process, due to deviations from the intended interventions (effect of assignment to intervention), in measurement of the outcome, in selection of the reported result as well as missing outcome data. The researchers resolved discrepancies by reaching consensus.

### Synthesis of results

The articles were summarized according to their setting (e.g. EMS, burn or trauma department). The objective was to present an overview of the psychological support recommended for or available to emergency patients in acute and emergency settings and to identify how healthcare professionals can assist in acute emergency situations.

## Results

### Selection of sources of evidence

A total of 1080 articles were identified in PubMed and PsycInfo databases, of which 14 were included, as illustrated in the PRISMA flowchart [45] below. Additionally, three relevant articles were identified through reference list checking, and an additional nine suitable articles were identified through forward citation searching. Consequently, 26 articles were included in the final analysis. The articles were published between the years 2003 and 2024.

### Characteristics of sources of evidence

A total of eight articles included in this study used qualitative methodologies [3, 4, 47–52], while nine used quantitative methodologies [53–61]. Amongst these, six were randomized controlled trials [55–59, 61]. One article was a systematic review [20], and another was a systematic review and a meta-analysis [21]. Finally, seven articles comprised narrative reviews [19, 62–67]. One article [50] has not been subjected to peer review.

The majority of articles were published in the USA (*n* = 7) [19, 21, 53, 55, 63, 64, 66], and Australia (*n* = 6) [4, 47, 48, 59, 62, 65]. A total of ten articles were published in Europe [3, 20, 50–52, 54, 56, 57, 60, 65]. The majority of interventions that have been tested or recommended are based on the perspective of healthcare systems in Europe, the USA and Australia.

### Critical appraisal within sources of evidence

The six included RCTs were assessed for risk of bias using the RoB 2 tool [46]. Three RCTs were judged to raise some concerns in at least one domain [55, 56, 61], three RCTs were judged to be at low risk of bias for all domains of this result [57–59], as illustrated in Table 2.

**Table 2:** Risk of bias assessment

	[55]	[56]	[57]	[58]	[59]	[61]
Randomization process	Low risk	Low risk	Low risk	Low risk	Low risk	Some concerns
Deviations from the intended intervention	Low risk	Some concerns	Low risk	Low risk	Low risk	Low risk
Missing outcome data	Low risk	Low	Low risk	Low risk	Low risk	Low risk
Measurement of outcome	Some concerns	Low risk	Low risk	Low risk	Low risk	Low risk
Selection of the reported result	Low risk	Low risk	Low risk	Low risk	Low risk	Low risk

### Results of individual sources of evidence

The articles addressed patients from a variety of emergency settings.^1^   The majority of studies focused on major and general trauma patients [4, 19, 49, 50, 53, 55, 56, 63, 64, 66]. The eligible articles investigated patients in the burn department [47, 48, 62], emergency rooms [20, 52, 57, 58], EMS [3, 51, 54], intensive care units (ICU) [60, 61, 67], as well as various departments such as general practitioners [65] and maternity wards [59].

The articles posit that the psychological consequences of emergency situations for patients are often overlooked [53] and call for further research into the provision of psychological support or interventions for emergency patients [19, 53, 63]. It is evident that there are feasible and effective interventions capable of assisting patients in the processing of their experience in the context of a medical emergency. These interventions have the potential to support the general sense of well-being and the reduction of psychological symptoms. A summary of the interventions can be found in tables 3, 4, 5, 6, 7, 8.

**Table 3:** Trauma department interventions

Reference	Intervention/Recommendation	Providers
Braaf 2018 [4]	Multimodal communication, consistent information provision and sharing, active communication, written discharge plans, presence of specialist trauma coordinator or trauma patient advocate	Healthcare professionals
Yadav & Shrestha 2017 [49]	Explaining and informing about amnesia, repeated preoperative education, building a trustworthy personnel-patient interaction	Healthcare professionals
Vincent 2015 [63]	Counselling, pastoral care, coping skills for pain, self-management, meditation and mindfulness, group support and networks, education and information about trauma and recovery	Pastor, clinical psychologist, healthcare professional
deRoon-Cassini 2019 [19]	Modified Prolonged Exposure (PE), Behavioural Activation (BA), stepped collaborative care, multitier approach to psychological intervention after traumatic-injury (MAPIT), screening	Psychologists, social workers, nurses, psychiatrists
Manser 2018 [55]	60-minute bedside consulting focusing on engagement, symptom education and normalization, emotional safety coping strategies, and an individualized referral, if desired, to a community mental health provider aiming to reduce initial distress and promote adaptive coping in the acute aftermath of trauma	Clinical coordinators, social work graduate research assistants
Tecic 2011 [56]	Inpatient psychotherapy: supportive and stabilizing elements, counselling, cognitive reorganization, imagination, resource activation, exposure, and relaxation techniques	Psychotherapist trained in psychotraumatology
Frank 2017 [53]	Primary Care-PTSD (PC-PTSD) Screening followed by a health psychology consult if risk for PTSD is confirmed	Nurses
Tanti 2023 [50]	Written information, journal, clear explanation of injuries using different modalities, two-way person centered conversations	Healthcare professionals
Obey & Miller 2022 [64]	Counselling and chaplain visits, client-centered-therapy, Trauma Collaborative Care program (TCC), Trauma Recovery Services (TRS) program, trauma-informed care (TIC), meditation and mindfulness, Trauma Survivors Network (TSN): peer visitation and family support	Orthopaedic surgeons and other healthcare professionals
Timmer-Murillo 2023 [66]	Trauma-informed care (TIC): modify communication to improve rapport and comfort, time for questions, involving patients in discussions on care, informing about procedures; screening of risk of psychopathology and reinjury; Psychological intervention: multitier approach to psychological intervention model, stepped-care approach (screening, features of CBT and motivational interviewing strategies)	Healthcare professionals

**Table 4:** Burn department interventions

Reference	Intervention/Recommendation	Providers
Gullick 2014 [47]	Available specialist psychologist with a proactive management strategy. Therapeutic approach for patients with facial burns. Burn patients and families should be engaged in discussions about possible emotional trauma, through written resources.	Psychologist, burn clinicians
Cleary 2020 [62]	TIC (trauma-informed care) 1. Before: Clinical-level chance, organisational integration, training and time 2. During: Creating safety, screening, collaboration with patient and supporters, use of peer-led services, mitigate risks of vicarious trauma 3. After: Proactive support through outreach and follow-up	Healthcare professionals
Johnson 2016 [48]	Early psychological support including psychological first aid, early discussions about emotional trauma, provision of written resources	Psychologist, burn clinicians

**Table 5:** Emergency medical service interventions

Reference	Intervention/Recommendation	Providers
Visser 2021 [3]	Informing patients about injury and treatment	Physicians and nurses
Arimon 2021 [54]	CONECTEM communicative intervention: augmentative alternative communication and basic communication skills adapted to the Glasgow Coma Scale.	EMS nurses
Nutbeam 2022 [51]	Positive communication and reassurance during the extraction of patients from a motor vehicle	Emergency worker, bystander

**Table 6:** Emergency department interventions

Reference	Intervention/Recommendation	Providers
Visser 2017 [20]	Psychoeducation, CBT, supportive counselling, hypnosis, psychoeducation in combination with psychotherapy, EMDR	Nurses
Gil-Jardiné 2018 [57]	Reassurance session (psychoeducation and cognitive distortions) and EMDR	ED healthcare professionals (therapist and psychologist)
Figueroa 2022 [58]	PFA-ABCDE: A: Active Listening B: Breathing retraining C: Categorization of needs D: *Derivación* (Referral) E. (psycho-) education	Psychology students with training in PFA-ABCDE
Willinge 2024 [52]	Humour, plain language, sharing medical images, a primarily responsible healthcare professional, clarity of diagnosis	ED healthcare professionals

**Table 7:** Intensive care unit interventions

Reference	Intervention/Recommendation	Providers
Peris 2011 [60]	Educational interventions, counselling, stress management, psychological support, coping strategies	Nurses, clinical psychologists
Mohta 2003 [67]	Informing patients, communication with respect Psychological interventions In case of amputation: counselling and psychotherapeutic interventions	ICU healthcare professionals
Cekic 2022 [61]	Additional information, supportive nursing care	Researchers

**Table 8:** Interventions in other departments

Reference	Intervention/Recommendation	Providers
Wade 2013 [65]	Stepped care approach: 1. Early response: advice and support, 2. Simple psychological strategies, 3. Formal mental health interventions	General practitioners
Gamble 2005 [59]	Counselling within 72 hours of birth: critical stress debriefing and issues relevant to the childbearing context	Midwifes
Gartlehner 2013 [21]	Debriefing, brief trauma-focused CBT, supportive counselling, collaborative care	Healthcare professionals

### Trauma department

Ten articles included in the review focused on trauma patients, particularly those who had experienced a major trauma [4, 19, 49, 50, 53, 55, 56, 63, 64, 66]. However, some traumatic injuries were not adequately described.

The provision of information [49] in a clear and reassuring manner [4, 50], the implementation of multi-modal communication strategies, such as additional written information and the presence of a specialist trauma coordinator or trauma patient advocate, have been identified as approaches that could prevent distress and insecurity among patients [4]. Education about trauma and recovery can assist patients in understanding their symptoms [63]. Humour should be employed when appropriate, and communication should be caring and helpful to improve patients’ care experience [50]. Healthcare professionals should assist patients in communicating with their family, friends, or colleagues [49]. It is important to respond to patients’ vulnerability, powerlessness, and dependency with empathy, kindness, and respect, and to encourage them to engage in their treatment [50].

Quantitative studies and narrative reviews have highlighted the importance of early identification of traumatic stress in the prevention of severe disorders [19, 53, 55, 56, 63, 64, 66].

The implementation of a PTSD screening tool can increase nurses’ awareness of patients’ psychological health, leading to better referrals and improved outcomes for patients [53]. Holistic, interdisciplinary care for trauma patients can assist them in coping and returning to a high quality of life [53]. However, a brief intervention for acute care trauma patients that focused on engagement, symptom education, coping strategies, and individualized referrals to mental health providers did not reduce or prevent the development of PTSD [55]. Ongoing psychotherapy sessions, which begin in the emergency room, have been demonstrated to reduce mental health symptoms, particularly anxiety symptoms [56]. However, the implementation of psychotherapy sessions within the ED does not seem to be feasible due to the potential for disruption and disturbance [56].

Narrative reviews have recommended various interventions with a positive impact on patients [19, 63, 64, 66]. These include trauma-informed care (TIC), as a more comprehensive understanding of potential traumas in patients among healthcare professionals, which can lead to improved patient care [64, 66]. Disorder specific interventions, such as modified prolonged exposure for PTSD and behavioural activation for depression, have demonstrated positive effects on mental health outcomes [19]. Stepped care approaches, such as the “multitier” approach to psychological intervention model, which comprises screening, consultation and further evaluation by the patient’s trauma psychology team, psychoeducation and interventions such as cognitive behavioural and motivational interviewing, are both feasible and effective in the identification and support of patients who could benefit from further intervention [19]. Furthermore, there is evidence that counselling and pastoral care have a positive impact on mental health [63, 64], including a reduction in anxiety, depression and PTSD symptoms [63]. Meditation has been demonstrated to assist patients in overcoming fear and emotions that may hinder recovery [63] as well as reducing symptoms of depression and PTSD [64].

### Burn department

Three of the included articles focused on burn injury patients [47, 48, 62]. In these articles is recommended that burn patients receive early psychological support and additional written information [47, 48].

Patients should be provided with psychological first aid, which includes physical and emotional comfort, perceived safety, and early information about possible emotional reactions to trauma [47]. Furthermore, it is important to encourage contact with family and friends [47, 48]. Additionally, it is recommended that a specialist psychologist should be available and proactively offer their services to patients and their family members [47].

Cleary et al. (2020) advocate for the implementation of TIC, consistent of the implementing of safety measures, the screening of patients, the involvement of patients in the intervention and recovery planning process, the provision of psychoeducation to patients and their supporters, and the utilisation of peer-led services [62]. The utilisation of TIC has the potential to reduce the psychological distress and harm associated with burn injuries, and to facilitate patient recovery in the short and long term [62].

### Emergency medical service (EMS)

Three articles were identified that provide information on the EMS, specifically critical care patients being transported by ambulance [54], the extraction of patients who were trapped in motor vehicles [51] and the transfer of patients to the ED by ambulance [3].

Patients require information about their injuries and treatment while being cared for by the ambulance and emergency room team [3, 51]. This becomes particular crucial in cases of memory loss [3]. The utilisation of the augmentative assistive communication intervention CONECTEM (catalan for ‘let’s connect’) during ambulance transport, which comprises augmentative alternative communication and basic communication skills adapted to the level of consciousness as measured by the Glasgow Coma Scale, resulted in a significant reduction in anxiety and PTSD symptoms (*p* < 0.001) [54]. Nutbeam et al. (2022) highlight the importance of communication for rescue teams performing extrications. It is recommended that an ‘extrication buddy’ be assigned to explain the procedure in clear and accessible language, provide companionship, and reassure the patient while trapped [51]. Information about the safety of co-occupants should be provided as soon as possible [51]. Furthermore, communication with family members should be facilitated [51].

### Emergency department

A total of four articles focused on the Emergency department (ED) [20, 52, 57, 58].

A single session of eye movement desensitisation and reprocessing recent traumatic episode protocol (EMDR R-TEP) psychotherapy, performed at the ED in the first hours after the traumatic event, reduces the rate of PTSD (*p* = 0.057) and post-concussion-like syndrome (PCLS) symptoms (*p* = 0.001) in patients with a high risk of PCLS compared to the control group [57]. This adapted EMDR protocol includes an additional reassurance session with psychoeducation and identification, discussion and challenging of any cognitive distortions [57].

Figueroa et al. (2022) conducted an RCT to examine the effectiveness of a psychological first aid intervention known as PFA-ABCDE (A = active listening, B = breathing retraining, C = categorization of needs, D = *Derivación* (referral) to social support networks, E = (psycho-) education). The intervention led to a higher level of immediate distress relief (Cohen’s d = 0.30, *p* = 0.038) and fewer PTSD symptoms at the one-month follow-up (Cohen’s d = 0.42, *p* = 0.033). However, no significant difference was observed in depressive symptoms at the one-month follow-up (*p* = 0.713) or PTSD symptoms at the six-month follow-up (*p* = 0.986) in comparison to a control group that received psychoeducation [58].

The recommendations put forward by orthopaedic trauma patients with the intention of improving the ED visit can be summarized as follows: healthcare professionals should anticipate the evolving information needs that will arise after the ED visit and they should engage patients at the earliest possible stage of the ED process in order to clarify care processes and shape expectations [52]. Furthermore, patients should be informed and involved in all stages of the treatment plan and the decision-making process across the entirety of the pathway [52]. In addition, healthcare professionals should utilize plain language and appropriate use of humour to reframe challenging situations [52]. Moreover, sharing and explaining medical images could aid in the comprehension of injuries [52]. Patients tend to prefer a single healthcare professional who is primarily responsible for their care, and clarity regarding their diagnoses [52].

In their systematic review, Visser et al. (2017) conclude that early treatment can prevent the development of PTSD. As a first step, it is recommended that nurses provide psychoeducation, with a referral for further treatment if necessary. A feasible stepped care intervention or cognitive behavioural therapy (CBT) elements could help overcome barriers. The review identified effective treatments as psychoeducation alone or combined with psychotherapy, CBT alone or combined with hypnosis and EMDR. Furthermore, the review identified that self-help booklets and internet-based CBT were ineffective [20].

### Intensive care unit

Three articles focused on the ICU were identified [60, 61, 67].

An observational study shows that early intra-ICU psychological intervention for trauma patients requiring mechanical ventilation can reduce the risk of PTSD, anxiety and depression at 12 months after ICU discharge. The interventions comprises of educational interventions, counselling, stress management, psychological support and coping mechanisms [60].

In a narrative review by Mohta et al. it is recommended that healthcare professionals should demonstrate their respect for patients through interventions such as maintaining eye contact and sitting down instead of standing when possible. Furthermore, personal bedside objects should be allowed at the bedside, the usual day-night cycle should be maintained whenever possible and family visits should be allowed as soon as possible. A comprehensive understanding of the situation reduces the feeling of anxiety, helplessness and sense of immobilisation for patients [67].

An RCT shows that providing information and supportive nursing care reduced anxiety, stress and agitation in COPD patients treated with non-invasive ventilation (NIV) [61]. The information about NIV was provided in person supported by a leaflet. Supportive care is defined as allowing patients to express their feelings and thoughts about NIV treatment, using an accepting and empathetic approach, therapeutic touch techniques, as well as explaining to patients how to reach the nurse when needed, arranging the environment, explaining each procedure with its objectives, use of coping strategies, and ensuring that patients are able to meet with their relatives during visiting hours [61].

### Other departments

Three articles from other departments were identified [21, 59, 65].

A counselling intervention, comprising elements of critical stress debriefing and issues pertinent to the childbearing context, delivered by midwives within 72 hours of birth, was found to effectively reduce symptoms of trauma (*p* = 0.035), postnatal depression (*p* = 0.002) depression (*p* = 0.005), stress (*p* = 0.029), and feelings of self-blame in postnatal women [59].

General practitioners should provide a sense of safety and self-efficacy, teach calming strategies, promote hope, help connect patients to social support, and monitor those who have experienced traumatic situations [65].

Gartlehner et al. (2013) conducted a systematic review and meta-analysis. For the majority of interventions studied in populations exposed to psychological trauma, no reliable evidence was found to support efficacy in preventing or reducing PTSD symptoms [21]. The findings demonstrated that brief trauma-focused CBT was more effective in reducing PTSD symptoms than supportive counselling [21]. It was demonstrated that collaborative care resulted in a greater reduction in PTSD symptoms for trauma patients requiring surgical hospitalization than usual care [21]. The evidence indicates that debriefing does not result in a reduction in PTSD symptoms or related psychological symptoms in civilian victims of crime, assault or accident trauma [21].

### Synthesis of results

In summary, the research situation and recommendations for the psychological treatment of emergency patients are highly diverse. Nevertheless, one factor is evident in almost all departments: patients require information and (psycho-)education from healthcare professionals to reduce their psychological distress and maintain or regain mental health.

The research evidence on the following interventions is inconclusive or demonstrates ineffectiveness: CBT based interventions have been shown to be effective in various reviews [20, 21, 66]. Nevertheless, a systematic review has demonstrated that internet-based CBT and self-help booklets are ineffective [20]. The provision of debriefing to patients is recommended as a component of an intervention in a randomized controlled trial [59], yet it is explicitly not advised in a narrative review [65] and a systematic review and meta-analysis [21].

## Discussion

### Summary of evidence

The objective of this scoping review was to present an overview of the psychological care recommended for or available to emergency patients and to identify how healthcare professionals can assist in acute emergency medicine situations. This review highlights the small and heterogeneous research landscape in psychological support for emergency patients across different healthcare settings. Except from the emphasis on educating and informing patients about injury mechanism, diagnosis, and treatment to reduce acute psychological distress and psychological symptoms, there is no consistent psychological intervention or communication strategy across the included studies.

One of the major findings of this scoping review is that 17 out of 26 included articles examined the impact of psychoeducation and of informing patients about their diagnosis and treatment. Both psychoeducation and informing are conducted by healthcare professionals of various professions and in all settings that have been included in this review. Overall, they have a positive effect on patients’ mental health by reducing psychological stress. Furthermore, patients claim that they need to be informed and educated by healthcare professionals. Being informed can lead to a new appraisal which may reduce stress [6]. Knowledge according to a mixed methods study of trauma exposed adults [75] can function as a coping mechanism in reducing perceived helplessness through the ability of anticipating further actions. Perceived helplessness as induced by a life event of major emotional significance, e. g. a traumatic event, can be an aetiological factor for generalized helplessness which can function as an aetiological as well as maintenance factor for depression [76]. Psychoeducation is generally effective in reducing stress, thereby making a positive contribution to mental health [77]. Research about psychoeducation after traumatic events is both limited and heterogeneous [78, 79]. Psychoeducation in the context of traumatic experiences serves to normalize stress responses and helps prevent patients from perceiving themselves as dysfunctional [79].

The heterogeneity of the research presented may be due to the increased focus on the psychological care of acute and emergency patients. For decades, the primary objective of emergency medicine has been the pure survival and best possible physical outcome of patients [80]. Notwithstanding the continued significance of this primary factor, as somatic medical care improves, the psychological consequences of the traumatic experience have been evidenced by the prevalence of psychological distress in patients receiving emergency healthcare. It is noteworthy that 19 out of the 25 included articles have been published within the last decade even though there was no restriction placed towards the publication date. This underlines the recently growing importance of research in emergency psychology.

The repercussions of inadequate psychological care have been brought to the fore by the Coronavirus disease 2019 (Covid-19) pandemic. During the pandemic, the social isolation of infected patients has resulted in a lack of social interaction [81]. Consequences for the patients were heightened psychological distress, such as panic after diagnosis, feelings of loneliness, helplessness, shame, anger and uncertainty [81, 82]. Covid-19 patients suffered from inadequate information about their disease and expressed desire for communication and emotional support [81], similar to the needs of the variety of emergency patients described in this review. Research about the psychological effects on Covid-19 patients calls for more attention to psychological changes in patients [81], identification of psychological consequences to ensure an adequate provision of services [83] as well as clear rational for decisions and information about procedures [84]. Due to media focus on psychological care of patients during the Covid-19 crisis [85, 86], we expect more research and knowledge about the psychological needs of critically ill patients.

Moreover, this review highlights the critical role of healthcare professionals, particularly nurses, in providing psychological support to emergency patients. Nurses play a crucial role in educating patients about their condition, offering emotional support, and facilitating communication between patients and their families [87]. Communication with emergency patients is often overlooked, yet it should be respectful, prompt, empathetic, open, caring, helpful, and reassuring.

Nevertheless, the global shortage of trained healthcare professionals and the high workload in the healthcare field may reduce the quality of care [88, 89]. Furthermore, the increasing number of international healthcare professionals [90] may potentially lead to language barriers that could compromise patient satisfaction, psychological care quality, and safety [91, 92]. Hence, additional training for healthcare staff and international colleagues addressing aspects of language and culture might be necessary. This underscores the significance for the research community to provide reliable, evidence-based communication strategies and psychological interventions. The combination of an excessive workload in time-critical emergency situations, a global shortage of trained healthcare professionals [88, 89] and the potential for language barriers [90–92] might place significant stress on professionals. Healthcare professionals may be reluctant to engage with additional research projects that involve alterations to workflow and an increase in workload, as these may exacerbate the existing challenges they face.

This review highlights a significant gap in psychological support for emergency patients across different healthcare settings. Despite numerous studies, there is still a lack of standardized evidence-based psychological interventions tailored to the specific needs of emergency patients. In addition, attention is called to the necessity for further research and standardized evidence-based interventions to address the psychological needs of emergency patients. Should the research continue to demonstrate heterogeneity in its effects on patients, it would be worthwhile to consider the patients’ individual reactions to traumatic events: it is possible that the diverse individual responses to trauma may impede the implementation of a universal standard approach. The implementation of a universal standard approach may also be affected by the range of clinical setting and patient conditions. It remains unclear from this review how easily the interventions could be applied in diverse emergency environments. However, it does illustrate the spectrum of psychological care that is currently available or that has the potential to be made available to patients. Common factor through the heterogeneous emergency healthcare is the provision of education and information to patients regarding injury mechanisms, diagnostic procedures, and treatment options to reduce acute psychological distress and psychological symptoms.

Future research should consider different patient conditions and could for instance adapt psychological care according to the patients Glasgow Coma Scale (GCS), as Arimon et al. (2021) suggest [54].

This review emphasizes the importance of collaboration between healthcare professionals, researchers, and policymakers to develop evidence-based guidelines and resources for the delivery of comprehensive psychological care in emergency healthcare settings. By prioritizing psychological support alongside medical treatment, healthcare systems can enhance the overall well-being and recovery of emergency patients, both physically and psychologically.

### Limitations

The aim of this scoping review was to present an overview of the psychological care recommended for or available to emergency patients and to identify how healthcare professionals can assist in acute emergency situations. Due to the heterogeneous nature of settings, we cannot provide an universal approach of best practice.

Additionally, a few limitations may have contributed to this outcome. Due to resource restrictions, we only searched two databases. In order to mitigate the consequences of a restricted search, a forward and backward citation search was conducted. The forward citation search yielded a considerable number of results, as illustrated in Fig. 1. This is typically indicative of a search term that requires enhancement. The Search Term Analyser [44] revealed the presence of two additional search terms which could have been incorporated into the original search query. However, a comparison of the original term with the extended term did not result in any changes to the results that would render them more relevant. Instead, the results became less suitable. Due to the heterogeneous research status and the ambiguity of the term “trauma”, it can be assumed that changing the search term would not have improved the results. It is therefore likely that the relevant articles were mainly found through the citation search. This is also supported by the comparisons conducted during the process of creating the search terms. Nevertheless, it is possible that a combination of search terms and boolean operators that were not identified by the research team may exist. It is estimated that this would not significantly affect the research outcomes.

Most studies (*n* = 12) report about general and major trauma patients. Only a few report about non-trauma related emergencies in settings such as maternity wards (*n* = 1) and emergency settings such as a general practitioner practices (*n* = 1). The small amount of studies outside of trauma patients is notable as patients experience psychological distress [93] and psychological diseases such as depression [94] or PTSD [95, 96] following non-trauma-related medical emergencies, including acute coronary syndrome or resuscitation [97].

The heterogeneous patient populations and medical settings might have impeded the process of providing a summary of communication strategies and interventions that healthcare professionals can use. Due to the restricted number of articles about psychological care in emergency settings, it was not possible to apply more homogeneous search restrictions.

The majority of articles are written from the perspective of health systems in Europe, USA and Australia. This facilitates the comparison process, but it is possible that in a different socio-cultural context, different psychological interventions and recommendations would be effective. The inclusion of articles written in languages other than English or German might have increased generalisability by offering a more nuanced perspective on socio-cultural values that are not solely representative of western values.

## Conclusion

Emergency healthcare professionals should focus on communicating effectively and empathically with patients. This review suggests that providing (psycho-) education and explanations regarding injury or illness and treatment can reduce psychological distress and subsequent impairments.

Given the heterogeneous nature of the research landscape, further research is recommended to identify distinct interventions for acute and emergency patients. A comprehensive investigation of patient needs is required. Healthcare professionals should be consulted regarding the possibility of implementing psychological support in emergency healthcare. Recommendations for psychological interventions and communication strategies should be verified by experts and pilot studies before starting confirmatory randomized-controlled trials to reduce the economic costs. Care should be taken to ensure that the interventions remain consistent in order to maintain comparability.

## Electronic supplementary material

Below is the link to the electronic supplementary material.

Supplementary Material 1

Supplementary Material 2

Supplementary Material 3

Supplementary Material 4

## Data Availability

All data generated or analysed during this study are included in this published article and its supplementary information files.

## Abbreviations

CBT: Cognitive behavioral therapy
DSM-5: Diagnostic and Statistical Manual of Mental Disorders Fifth Edition
ED: Emergency department
EMS: Emergency Medical Service
EMT: Emergency Medical Technician
GCS: Glasgow Coma Scale
ICU: Intensive care unit
NIV: Non-invasive ventilation
PCC: Population, Concept, Context
PRISMA-ScR: Preferred Items for Systematic Reviews and Meta-Analysis extension for Scoping Reviews
PRISMA: Preferred Items for Systematic Reviews and Meta-Analysis
PTSD: Posttraumatic stress disorder
RCT: Randomized controlled trial
ROB 2: Risk of Bias 2 tool
SRA: Systematic Review Accelerator
TIC: Trauma informed care
TSN: Trauma survivor network

## Footnotes

1 The emergency settings investigated are illustrated in the supplementary material 4 [68, 69, 70, 71, 72, 73, 74].
