# Supplementary material for: Psychological care in acute and emergency medicine: a scoping review of support interventions by healthcare professionals
Source: BMC Emerg Med. 2026 Feb 18;26:64. doi: 10.1186/s12873-026-01494-y (PMC12930831; doi:10.1186/s12873-026-01494-y)
Supplement: Supplementary file 1 — Supplementary Material 1 [file 12873_2026_1494_MOESM1_ESM.pdf]

Supplementary material 1: List of excluded articles read in full text

Reference	Reason of exclusion				
	<18	Nft	R1	R2	R3
Abass G, Asery A, Al Badr A, AlMaghlouth A, AlOtaiby S, Heena H. Patient satisfaction with the emergency department services at an academic teaching hospital. J Family Med Prim Care. 2021;10:1718.				X	
Abdelraof AI, Sliman AMA, Ibrahim AA-W, El-Etreby RR. Relationship between Psychosocial Care and ICU Trauma among Patients underwent Open-Heart Surgery. 2022;13.				X	
Alimohammadi N, Ziaeirad M, Irajpour A, Aminmansour B. Clinical Care Needs of Patients with Severe Traumatic Brain Injury in the Intensive Care Unit. Trauma Mon. 2018;23.				X	
Archer KR, Davidson CA, Alkhoury D, Vanston SW, Moore TL, Deluca A, et al. Cognitive-Behavioral–Based Physical Therapy for Improving Recovery After Traumatic Orthopaedic Lower Extremity Injury (CBPT-Trauma). Journal of Orthopaedic Trauma. 2022;36:S1–7.					X
Batt RJ. Empirical Studies In Hospital Emergency Departments. 2013.			X		
Bayuo J, Wong FKY, Agyei FB. “On the Recovery Journey:” An Integrative Review of the Needs of Burn Patients From Immediate Pre-Discharge to Post-Discharge Period Using the Omaha System. J of Nursing Scholarship. 2020;52:360–8.					X
Beaton A, O’Leary K, Thorburn J, Campbell A, Christey G. Improving patient experience and outcomes following serious injury. N Z Med J. 2019;132:15–25.				X	
Bertelson A, Brasel KJ, deRoon-Cassini TA. Implementing a Posttraumatic Stress and Functional Outcome Screening Process for Trauma Patients at a Level 1 Adult Trauma Center. Journal of Trauma Nursing. 2011;18:5–8.				X	
Bérubé M, Bradley N, O’Donnell M, Stelfox HT, Garraway N, Vasiliadis H-M, et al. Clinical practice guideline recommendations to improve the mental health of adult trauma patients: protocol for a systematic review. BMJ Open. 2024;14:e079205.		X			
Beutel A, Sanftenberg L, Friemel CM, Kosilek RP, Schauer M, Elbert T, et al. Patient perspectives on stress after ICU and a short primary care based psychological intervention – results from a qualitative sub study of the PICTURE trial. BMC Prim Care. 2025;26:12.					X
Bidhendi S, Ahmadi A, Fouladinejad M, Bazargan-Hejazi S. Evaluating implementation of WHO Trauma Care Checklist vs. modified WHO checklist in improving trauma patient clinical outcomes and satisfaction. J Inj Violence Res. 2021;13:5–12.			X		

Reference	Reason of exclusion				
	<18	Nft	R1	R2	R3
Blackburn J, Ousey K, Goodwin E. Information and communication in the emergency department. International Emergency Nursing. 2019;42:30–5			X		
Bliton JN, Zakrison TL, Vong G, Johnson DA, Rattan R, Hanos DS, et al. Ethical Care of the Traumatized: Conceptual Introduction to Trauma-Informed Care for Surgeons and Surgical Residents. Journal of the American College of Surgeons. 2022;234:1238–47.			X		
Blumenfield M, Schoeps M. Psychological Care of the Burn and Trauma Patient. Br J Psychiatry. 1993;163:283–283.				X	
Boudreaux ED, O'Hea EL. Patient satisfaction in the Emergency Department: a review of the literature and implications for practice. The Journal of Emergency Medicine. 2004;26:13–26.			X		
Bradbury E. Meeting the psychological needs of patients with facial disfigurement. British Journal of Oral and Maxillofacial Surgery. 2012;50:193–6.					X
Brunet A, Des Groseilliers IB, Cordova MJ, Ruzek JI. Randomized controlled trial of a brief dyadic cognitive-behavioral intervention designed to prevent PTSD. European Journal of Psychotraumatology. 2013;4:21572.					X
Bryant RA, Harvey AG, Dang ST, Sackville T, Basten C. Treatment of acute stress disorder: A comparison of cognitive-behavioral therapy and supportive counseling. Journal of Consulting and Clinical Psychology. 1998;66:862–6.					X
Bryant RA, Moulds ML, Guthrie RM, Nixon RDV. The Additive Benefit of Hypnosis and Cognitive-Behavioral Therapy in Treating Acute Stress Disorder. Journal of Consulting and Clinical Psychology. 2005;73:334–40.					X
Bryant RA, Moulds ML, Nixon RVD. Cognitive behaviour therapy of acute stress disorder: a four-year follow-up. Behaviour Research and Therapy. 2003;41:489–94.					X
Bugg A, Turpin G, Mason S, Scholes C. A randomised controlled trial of the effectiveness of writing as a self-help intervention for traumatic injury patients at risk of developing post-traumatic stress disorder. Behaviour Research and Therapy. 2009;47:6–12.					X
Bulger EM, Johnson P, Parker L, Moloney KE, Roberts MK, Vaziri N, et al. Nationwide Survey of Trauma Center Screening and Intervention Practices for Posttraumatic Stress Disorder, Firearm Violence, Mental Health, and Substance Use Disorders. Journal of the American College of Surgeons. 2022;234:274–87.				X	
Bunnell BE, Davidson TM, Winkelmann JR, Maples-Keller JL, Ridings LE, Dahne J, et al. Implementation and Utility of an Automated Text Messaging System to Facilitate Symptom Self-Monitoring and Identify Risk for Post-Traumatic Stress Disorder and Depression in Trauma Center Patients. Telemedicine and e-Health. 2019;25:1198–206.				X	

Reference	Reason of exclusion				
	<18	Nft	R1	R2	R3
C Wong E, N Marshall G, Nv Miles J. Randomized Controlled Trial of a Psychoeducational Video Intervention for Traumatic Injury Survivors. J Trauma Stress Disor Treat. 2013;02.				X	
Calder A, Badcoe A, Harms L. Broken bodies, healing spirits: road trauma survivor's perceptions of pastoral care during inpatient orthopaedic rehabilitation. Disability and Rehabilitation. 2011;33:1358–66.					X
Carlson EB, Spain DA, Muhtadie L, McDade-Montez L, Macia KS. Care and caring in the intensive care unit: Family members' distress and perceptions about staff skills, communication, and emotional support. J Crit Care. 2015;30:557–61.				X	
Chandra A, Marshall GN, Shetty V, Paddock SM, Wong EC, Zatzick D, et al. Barriers to seeking mental health care after treatment for orofacial injury at a large, urban medical center: concordance of patient and provider perspectives. J Trauma. 2008;65:196–202.				X	
Ching THW, Gitajn IL, Rotenberg S, Lyons KD, Brady RE. Behavioral Activation for Orthopedic Trauma Patients After Discharge: A Case Series Report of an Open-Label, Prospective Observational Pilot Study. Cognitive and Behavioral Practice. 2023;30:597–609.					X
Chow J, Lancman B. Psychiatric sequelae and interventions in critically ill trauma survivors. Curr Opin Anaesthesiol. 2023;36:147–52.			X		
Cinnella G, Gambatesa, D'Antini, Mirabella, De Capraris, Iuso S, et al. Counseling, quality of life, and acute postoperative pain in elderly patients with hip fracture. JMDH. 2013;:335.					X
Cleary M, West S, McGarry D, Kornhaber R. Self-Immolation and the Mental Health Care of Survivors. Issues in Mental Health Nursing. 2020;41:655–7.			X		
Condon N, Kindelan KM. The Emotional Care of a Person With a Spinal Cord Injury. Journal of the American Medical Association. 1984					X
Cook M, Zonies D, Brasel K. Prioritizing Communication in the Provision of Palliative Care for the Trauma Patient. Curr Trauma Rep. 2020;6:183–93.			X		
Creer TL, Reynolds RV, Kotses H. Psychological Theory, Assessment, and Interventions for Adult and Childhood Asthma. In: Sweet JJ, Rozensky RH, Tovian SM, editors. Handbook of Clinical Psychology in Medical Settings. Boston, MA: Springer US; 1991. p. 497–515.	X				
Damböck M. Geriatric Education for Emergency Medical Services: Der geriatrische Patient im Rettungsdienst. Z Gerontol Geriat. 2021;54:99–105.			X		

Reference	Reason of exclusion				
	<18	Nft	R1	R2	R3
Darnell DA, Parker LE, Wagner AW, Dunn CW, Atkins DC, Dorsey S, et al. Task-shifting to improve the reach of mental health interventions for trauma patients: findings from a pilot study of trauma nurse training in patient-centered activity scheduling for PTSD and depression. Cognitive Behaviour Therapy. 2019;48:482–96.			X		
Darnell D, O'Connor S, Wagner A, Russo J, Wang J, Ingraham L, et al. Enhancing the Reach of Cognitive-Behavioral Therapy Targeting Posttraumatic Stress in Acute Care Medical Settings. PS. 2017;68:258–63.					X
Davidson TM, Espeleta HC, Ridings LE, Witcraft S, Bravoco O, Higgins K, et al. Implementation of a Stepped Care Program to Address Emotional Recovery among Traumatic Injury Patients. Journal of the American College of Surgeons. 2023;237:810–25.			X		
Des Groseilliers IB, Marchand A, Cordova MJ, Ruzek JI, Brunet A. Two-year follow-up of a brief dyadic cognitive-behavioral intervention designed to prevent PTSD. Psychological Trauma: Theory, Research, Practice, and Policy. 2013;5:462–9.					X
Ding L, Tao X, Zhou J. Effect of a comprehensive geriatric assessment-based individualized intervention on postoperative patients with cerebral hemorrhage: A randomized controlled study. Technol Health Care. 2024;32:1555–67.			X		
Dziadzko V, Dziadzko MA, Johnson MM, Gajic O, Karnatovskaia LV. Acute psychological trauma in the critically ill: Patient and family perspectives. General Hospital Psychiatry. 2017;47:68–74.			X		
Elmqvist C, Fridlund B, Ekebergh M. More than medical treatment: The patient's first encounter with prehospital emergency care. International Emergency Nursing. 2008;16:185–92.				X	
Epperson MM. Families In Sudden Crisis: Process And Intervention In A Critical Care Center. Social Work in Health Care. 1977;2:265–73.					X
Ewens BA, Hendricks JM, Sundin D. Never ending stories: visual diarizing to recreate autobiographical memory of intensive care unit survivors. Nursing in Critical Care. 2017;22:8–18.					X
Fakhry SM, Ferguson PL, Olsen JL, Haughney JJ, Resnick HS, Ruggiero KJ. Continuing Trauma: The Unmet Needs of Trauma Patients in the Postacute Care Setting. Am Surg. 2017;83:1308–14.			X		
Fauman BJ. Psychiatric residency training in the management of emergencies. Psychiatr Clin North Am. 1983;6:325–34.					X
Forneris CA, Gartlehner G, Brownley KA, Gaynes BN, Sonis J, Coker-Schwimmer E, et al. Interventions to Prevent Post-Traumatic Stress Disorder. American Journal of Preventive Medicine. 2013;44:635–50.					X

Reference	Reason of exclusion				
	<18	Nft	R1	R2	R3
Gagliardi AR, Boyd JM, Evans D, Gerein L, Nathens A, Stelfox HT. Establishing components of high-quality injury care: Focus groups with patients and patient families. Journal of Trauma and Acute Care Surgery. 2014;77:749–56.				X	
Galatzer-Levy IR, Ankri Y, Freedman S, Israeli-Shalev Y, Roitman P, Gilad M, et al. Early PTSD Symptom Trajectories: Persistence, Recovery, and Response to Treatment: Results from the Jerusalem Trauma Outreach and Prevention Study (J-TOPS). PLoS ONE. 2013;8:e70084.					X
Gardner AJ, Griffiths J. Propranolol, post-traumatic stress disorder, and intensive care: incorporating new advances in psychiatry into the ICU. Crit Care. 2014;18:698.					X
Garrouste-Orgeas M, Flahault C, Fasse L, Ruckly S, Amdjar-Badidi N, Argaud L, et al. The ICU-Diary study: prospective, multicenter comparative study of the impact of an ICU diary on the wellbeing of patients and families in French ICUs. Trials. 2017;18:542.					X
Gironda MW, Der-Martirosian C, Belin TR, Black EE, Atchison KA. Predictors of Depressive Symptoms Following Mandibular Fracture Repair. Journal of Oral and Maxillofacial Surgery. 2009;67:328–34.				X	
Giummarra MJ, Lennox A, Dali G, Costa B, Gabbe BJ. Early psychological interventions for posttraumatic stress, depression and anxiety after traumatic injury: A systematic review and meta-analysis. Clinical Psychology Review. 2018;62:11–36.					X
Glimelius Petersson C, Ringdal M, Apelqvist G, Bergbom I. Diaries and memories following an ICU stay: a 2-month follow-up study. Nursing in Critical Care. 2018;23:299–307.					X
Goans CRR, Meltzer KJ, Martin B, Roaten K. Treatment Adherence Interventions for Burn Patients: What Works and What Role Can Motivational Interviewing Play? EBJ. 2022;3:309–19.					X
Goldman LS, Kimball CP. Depression in Intensive Care Units. Int J Psychiatry Med. 1988;17:201–12.					X
Gray MJ, Maguen S, Litz BT. Acute Psychological Impact of Disaster and Large-Scale Trauma: Limitations of Traditional Interventions and Future Practice Recommendations. Prehosp Disaster med. 2004;19:64–72.					X
Green BL, Kaltman S, Frank L, Glennie M, Subramanian A, Fritts-Wilson M, et al. Primary care providers' experiences with trauma patients: A qualitative study. Psychological Trauma: Theory, Research, Practice, and Policy. 2011;3:37–41.			X		
Guest E, Griffiths C, Harcourt D. A qualitative exploration of psychosocial specialists' experiences of providing support in UK burn care services. Scars, Burns & Healing. 2018;4:205951311876488.			X		

Reference	Reason of exclusion				
	<18	Nft	R1	R2	R3
Haagsma JA, Spronk I, De Jongh MAC, Bonsel GJ, Polinder S. Conventional and retrospective change in health-related quality of life of trauma patients: an explorative observational follow-up study. Health Qual Life Outcomes. 2020;18:157.				X	
Hansen E. Touching the unconscious in the unconscious – hypnotic communication with unconscious patients. Front Psychol. 2024;15:1389449.				X	
Harbin A. Prescribing Posttraumatic Growth. Bioethics. 2015;29:671–9.				X	
Harorani M, Davodabady F, Masmouei B, Barati N. The effect of progressive muscle relaxation on anxiety and sleep quality in burn patients: A randomized clinical trial. Burns. 2020;46:1107–13.	X				
Hobfoll SE, Watson P, Bell CC, Bryant RA, Brymer MJ, Friedman MJ, et al. Five Essential Elements of Immediate and Mid–Term Mass Trauma Intervention: Empirical Evidence. Psychiatry: Interpersonal and Biological Processes. 2007;70:283–315.			X		
Holeva V, Tarrier N, Wells A. Prevalence and predictors of acute stress disorder and PTSD following road traffic accidents: Thought control strategies and social support. Behavior Therapy. 2001;32:65–83.				X	
Huang AK, Campbell P-A, Chaudhary MJ, Soklaridis S, Miller D, Dinizulu S, et al. “We’re playing on the same team”: Communication (dis)connections between trauma patients and surgical residents. J Trauma Acute Care Surg. 2023;94:93–100.				X	
Hull AM, Lowe T, Devlin M, Finlay P, Koppel D, Stewart AM. Psychological consequences of maxillofacial trauma: a preliminary study. British Journal of Oral and Maxillofacial Surgery. 2003;41:317–22.				X	
Hung YW, Gallo JJ, Tol W, Syokau R, Bachani AM. Distress and resilience among unintentional injuries survivors in Kenya: A qualitative study. Rehabilitation Psychology. 2020;65:45–53.				X	
Hwang SH, Lim J. Evaluating the Effects of a Trauma Recovery Program for Korean Burn Patients. Res Soc Work Pract. 2021;31:493–502.					X
Jaramillo S, Suffoletto B, Callaway C, Pacella-LaBarbara M. Early Screening for Posttraumatic Stress Disorder and Depression Among Injured Emergency Department Patients: A Feasibility Study. Academic Emergency Medicine. 2019;26:1232–44.				X	
Jotzo M, Poets CF. Helping Parents Cope With the Trauma of Premature Birth: An Evaluation of a Trauma-Preventive Psychological Intervention. Pediatrics. 2005;115:915–9.					X
Kang KK, Ciminero ML, Parry JA, Mauffrey C. The Psychological Effects of Musculoskeletal Trauma. J Am Acad Orthop Surg. 2021;29:e322–9.				X	

Reference	Reason of exclusion				
	<18	Nft	R1	R2	R3
Karabatzakis M, Den Oudsten BL, Gosens T, De Vries J. Psychometric properties of the psychosocial screening instrument for physical trauma patients (PSIT). Health Qual Life Outcomes. 2019;17:172.				X	
Karnatovskaia LV, Varga K, Niven AS, Schulte PJ, Mujic M, Gajic O, et al. A pilot study of trained ICU doulas providing early psychological support to critically ill patients. Crit Care. 2021;25:446.					X
Kaufman EJ, Whitehorn G, Orji W, Chreiman K, Jackson S, Holena D, et al. Patient Experiences of Acute and Postacute Care After Trauma. Journal of Surgical Research. 2023;291:303–12.				X	
Kellezi B, Earthy S, Sleney J, Beckett K, Barnes J, Christie N, et al. What can trauma patients’ experiences and perspectives tell us about the perceived quality of trauma care? a qualitative study set within the UK National Health Service. Injury. 2020;51:1231–7.	X				
Kellezi B, Beckett K, Earthy S, Barnes J, Sleney J, Clarkson J, et al. Understanding and meeting information needs following unintentional injury: Comparing the accounts of patients, carers and service providers. Injury. 2015;46:564–71.			X		
Keough VA. Characteristics and Perceptions of Trauma Recidivists and Non-Recidivists.				X	
Kirven JC, Everhart JS, DiBartola AC, Jones J, Flanigan DC, Harrison R. Interventional Efforts to Reduce Psychological Distress After Orthopedic Trauma: A Systematic Review. HSS Jrnl. 2020;16:250–60.	X				
Kleve L, Robinson E. A survey of psychological need amongst adult burn-injured patients. Burns. 1999;25:575–9.	X				
Klewer J, Knopp W, Kugler J. Einsatz des Nottingham Health Profile zur Evaluation der Lebensqualität bei Patienten nach Behandlung einer offenen Unterschenkelfraktur.				X	
Knol R, Kelly E, Paul E, Cleland H, Wellington-Boyd A, Lambert C, et al. The psychosocial complexities of acute burn patients in an Australian trauma hospital. Burns. 2020;46:447–53.				X	
Knutzen T, Bulger E, Iles-Shih M, Hernandez A, Engstrom A, Whiteside L, et al. Stepped collaborative care versus American College of Surgeons Committee on Trauma required screening and referral for posttraumatic stress disorder: Clinical trial protocol. Contemporary Clinical Trials. 2024;136:107380.		X			
Kornfeld DS. Psychiatric view of the intensive care unit. BMJ. 1969;1:108–10.					X
Kornhaber R, McLean L, Rogers V, Proctor M-T, Kwiet J, Streimer J, et al. The patient–body relationship and the “lived experience” of a facial burn injury: a phenomenological inquiry of early psychosocial adjustment. JMDH. 2015;:377.				X	
Kotfis K, Van Diem-Zaal I, Williams Roberson S, Sietnicki M, Van Den Boogaard M, Shehabi Y, et al. The future of intensive care: delirium should no longer be an issue. Crit Care. 2022;26:200.					X

Reference	Reason of exclusion				
	<18	Nft	R1	R2	R3
Kuza C, Sheski D, Jones K. The Impact of a Multifaceted Delirium Preventing Strategy in the ICU involving Psychiatry: Effects on Delirium Incidence and Outcomes. 2018.		X			
Lampropoulou A, Hatzichristou C, Tadaros S. Psychological Support in Times of Crisis and Natural Disasters. In: Pikoulis E, Doucet J, editors. Emergency Medicine, Trauma and Disaster Management. Cham: Springer International Publishing; 2021. p. 555–61.			X		
Li A, Du F, Jin Y, Zhuang L. Clinical Evaluation of Comfort Nursing in Gynecological Patients Undergoing Laparoscopic Surgery. Altern Ther Health Med. 2023;29:311–5.					X
Lin C-S, Hsu M-YF, Chong C-F. Differences between emergency patients and their doctors in the perception of physician empathy: implications for medical education. Educ Health (Abingdon). 2008;21:144.				X	
Louw A, Diener I, Butler DS, Puentedura EJ. The Effect of Neuroscience Education on Pain, Disability, Anxiety, and Stress in Chronic Musculoskeletal Pain. Archives of Physical Medicine and Rehabilitation. 2011;92:2041–56.				X	
Maynard DC. Stages of Change in Drug-using Trauma Patients. 2016.			X		
McDermott L, Hotton M, Cartwright A. Understanding the Barriers and Enablers for Seeking Psychological Support Following a Burn Injury. EBJ. 2023;4:303–18.					X
McGraw C, Pekarek J, Redmond D, Vogel R, Tanner A, Bar-Or D. Is preexisting mental illness associated with lower patient satisfaction for older trauma patients? A cross-sectional descriptive study. BMC Psychiatry. 2021;21:67.				X	
McLean L, Chen R, Kwiet J, Streimer J, Vandervord J, Kornhaber R. A clinical update on posttraumatic stress disorder in burn injury survivors. Australas Psychiatry. 2017;25:348–50.					X
Moonilal JM. Trauma Centers, Social Work in Health Care. Social Work in Health Care. 1982;7:15–25.			X		
Moran ME. Health And Well-Being Of Physical Trauma Survivors: Who Follows Up? 2018.				X	
Morse JM, Proctor A. Maintaining Patient Endurance: The Comfort Work of Trauma Nurses. Clin Nurs Res. 1998;7:250–74.	X				
Mouthaan J, Sijbrandij M, De Vries G-J, Reitsma JB, Van De Schoot R, Goslings JC, et al. Internet-Based Early Intervention to Prevent Posttraumatic Stress Disorder in Injury Patients: Randomized Controlled Trial. J Med Internet Res. 2013;15:e165.				X	
Muntlin Å, Gunningberg L, Carlsson M. Patients' perceptions of quality of care at an emergency department and identification of areas for quality improvement. Journal of Clinical Nursing. 2006;15:1045–56.				X	
Naughton C, de Foubert M, Cummins H, McCullagh R, Wills T, Skelton DA, et al. Implementation of a Frailty Care Bundle (FCB) Targeting Mobilisation, Nutrition and Cognitive Engagement to Reduce Hospital Associated		X			

Reference	Reason of exclusion				
	<18	Nft	R1	R2	R3
Decline in Older Orthopaedic Trauma Patients: Pretest-Posttest Intervention Study. J Frailty Sarcopenia Falls. 2024;9:32–50.					
O'Donnell ML, Lau W, Tipping S, Holmes ACN, Ellen S, Judson R, et al. Stepped early psychological intervention for posttraumatic stress disorder, other anxiety disorders, and depression following serious injury. J Traum Stress. 2012;25:125–33.					X
Olive P, Hives L, Ashton A, O'Brien M, Taylor A, Mercer G, et al. Psychological and psychosocial aspects of major trauma care: A survey of current practice across UK and Ireland. Trauma. 2023;:146040862211455.			X		
Ough JK, Nampiaparampil DE. Pain After Traumatic Brain Injury. In: Moore RJ, editor. Handbook of Pain and Palliative Care. New York, NY: Springer New York; 2013. p. 177–94.				X	
Paniagua AR, Mundy LR, Klassen A, Biswas S, Hollenbeck ST, Pusic AL, et al. Resilience through practicing acceptance: A qualitative study of how patients cope with the psychosocial experiences following limb-threatening lower extremity trauma. J Plast Reconstr Aesthet Surg. 2022;75:3722–31.					X
Papathanassoglou ED. Psychological support and outcomes for ICU patients. Nursing in Critical Care. 2010;15:118–28.					X
Parker AM, Sricharoenchai T, Needham DM. Early Rehabilitation in the Intensive Care Unit: Preventing Impairment of Physical and Mental Health. Curr Phys Med Rehabil Rep. 2013;1:307–14.					X
Pham CH, Fang M, Nager J, Matsushima K, Inaba K, Kuza CM. The role of psychological support interventions in trauma patients on mental health outcomes: A systematic review and meta-analysis. J Trauma Acute Care Surg. 2019;87:463–82.	X				
Potter M, Aaron D, Mumford R, Ward L. An evaluation of clinical psychology input into burns multidisciplinary follow-up clinics. Scars, Burns & Healing. 2023;9:205951312211410.					X
Prater L, Bulger E, Maier RV, Goldstein E, Thomas P, Russo J, et al. Emergency Department and Inpatient Utilization Reductions and Cost Savings Associated With Trauma Center Mental Health Intervention: Results From a 5-year Longitudinal Randomized Clinical Trial Analysis. Annals of Surgery. 2024;279:17–23.	X				
Qi G, Li X, Yuan P, Chu X, Dai X, Shi X. Influencing factors and early predictive model of acute stress disorder in traumatic patients: A clinical comparative cohort study. Injury. 2024;55:111578.				X	
Rayamajhi S. Counselling support for critically ill patients and their families following a critical care experience: A qualitative study.					X
Resnick H, Acierno R, Holmes M, Kilpatrick DG, Jager N. Prevention of Post-Rape Psychopathology. Journal of Anxiety Disorders. 1999;13:359–70.				X	

Reference	Reason of exclusion				
	<18	Nft	R1	R2	R3
Rhodes A, Wilson C, Zelenkov D, Adams K, Poyant JO, Han X, et al. "The Psychiatric Domain of Post-Intensive Care Syndrome: A Review for the Intensivist." J Intensive Care Med. 2024;:08850666241275582.					X
Richards-Belle A, Mouncey PR, Wade D, Brewin CR, Emerson LM, Grieve R, et al. Psychological Outcomes following a nurse-led Preventative Psychological Intervention for critically ill patients (POPPI): protocol for a cluster-randomised clinical trial of a complex intervention. BMJ Open. 2018;8:e020908.		X			
Richter JC, Waydhas C, Pajonk F-G. Incidence of Posttraumatic Stress Disorder After Prolonged Surgical Intensive Care Unit Treatment. Psychosomatics. 2006;47:223–30.				X	
Robinson LJ, Stephens NM, Wilson S, Graham L, Hackett KL. Conceptualizing the key components of rehabilitation following major musculoskeletal trauma: A mixed methods service evaluation. Evaluation Clinical Practice. 2020;26:1436–47.					X
Rosendahl, Jenny, Reck, Johannes, Gawlytta, Romina, Böttche, Maria, Niemeyer, Helen, Glaesmer, Heide, et al. Belastungsfaktoren von Patient:innen und deren Angehörigen während -intensivmedizinischer Behandlung. Trauma & Gewalt. 2025;:58–69.		X			
Rothbaum BO, Kearns MC, Price M, Malcoun E, Davis M, Ressler KJ, et al. Early Intervention May Prevent the Development of Posttraumatic Stress Disorder: A Randomized Pilot Civilian Study with Modified Prolonged Exposure. Biological Psychiatry. 2012;72:957–63.					X
Ruggiero KJ, Anton MT, Davidson TM, deRoon-Cassini TA, Hink AB. It is time to prioritize complete trauma care. J Trauma Acute Care Surg. 2022;92:e18–21.				X	
Ruggiero KJ, Davidson TM, Anton MT, Bunnell B, Winkelmann J, Ridings LE, et al. Patient Engagement in a Technology-Enhanced, Stepped-Care Intervention to Address the Mental Health Needs of Trauma Center Patients. Journal of the American College of Surgeons. 2020;231:223–30.					X
Samim A, Rahimi-Bashar F, Gohari-Moghadam K, Izadi M, Karimi L, Jarineshin H, et al. Spouses as trained ICU doulas have significant effects on the psychological outcomes of intensive care survivors: A retrospective secondary analysis. preprint. In Review; 2022.				X	
Sandström L, Engström Å, Nilsson C, Juuso P. Experiences of suffering multiple trauma: A qualitative study. Intensive and Critical Care Nursing. 2019;54:1–6.				X	
Schmidt B, Deffner T, Rosendahl J. Feeling Safe during Intensive Care: Protocol of a Pilot Study on Therapeutic Suggestions of Safety under Hypnosis in Patients with Non-Invasive Ventilation. OBM ICM. 2020;5:1–8.		X			

Reference	Reason of exclusion				
	<18	Nft	R1	R2	R3
Scholes C, Turpin G, Mason S. A randomised controlled trial to assess the effectiveness of providing self-help information to people with symptoms of acute stress disorder following a traumatic injury. Behaviour Research and Therapy. 2007;45:2527–36.					X
Skogstad L, Hem E, Sandvik L, Ekeberg O. Nurse-Led Psychological Intervention After Physical Traumas: A Randomized Controlled Trial. J Clin Med Res. 2015;7:339–47.					X
Sen S, Palmieri T, Greenhalgh D. Review of Burn Research for Year 2014. Journal of Burn Care & Research. 2015;36:587–94.				X	
Sharma AK, Elbuluk AM, Gkiatas I, Kim JM, Sculco PK, Vigdorchik JM. Mental Health in Patients Undergoing Orthopaedic Surgery: Diagnosis, Management, and Outcomes. JBJS Reviews. 2021;9.					X
Shulman NM. A model of crisis intervention in critical and intensive care units of general hospitals. In: Yeager KR, Roberts AR, editors. Crisis intervention handbook: Assessment, treatment, and research., 4th ed. New York, NY: Oxford University Press; 2015. p. 658–77.					X
Silander NC, Chesire DJ, Scott KS. Psychological Prophylaxis: An Integrated Psychological Services Program in Trauma Care. J Clin Psychol Med Settings. 2019;26:291–301.			X		
Simske NM, Rivera T, Breslin MA, Hendrickson SB, Simpson M, Kalina M, et al. Implementing psychosocial programming at a level 1 trauma center: results from a 5-year period. Trauma Surg Acute Care Open. 2020;5:e000363.				X	
Sinkler MA, Furdock RJ, Vallier HA. Treating trauma more effectively: A review of psychosocial programming. Injury. 2022;53:1756–64.				X	
Skinner HK, Rahtz E, Korszun A. Interviews following physical trauma: A thematic analysis. Int Emerg Nurs. 2019;42:19–24.				X	
Sleney J, Christie N, Earthy S, Lyons RA, Kendrick D, Towner E. Improving recovery—Learning from patients' experiences after injury: A qualitative study. Injury. 2014;45:312–9.	X				
Sonis JD, White BA. Optimizing Patient Experience in the Emergency Department. Emergency Medicine Clinics of North America. 2020;38:705–13.			X		
Stevens TJ, Lee DB. Postintensive Care Syndrome: Feasibly Bridging Care at a Tertiary Trauma Center. J Trauma Nurs. 2023;30:242–8.			X		
Strain JJ. Psychological interventions in medical practice. New York: Appleton-Century-Crofts; 1978.					X
Sweet JJ, Rozensky RH, Tovian SM. Clinical Psychology in Medical Settings. In: Sweet JJ, Rozensky RH, Tovian SM, editors. Handbook of Clinical Psychology in Medical Settings. Boston, MA: Springer US; 1991. p. 3–9.					X

Reference	Reason of exclusion				
	<18	Nft	R1	R2	R3
Tan Y, Gajic O, Schulte PJ, Clark MM, Philbrick KL, Karnatovskaia LV. Feasibility of a Behavioral Intervention to Reduce Psychological Distress in Mechanically Ventilated Patients. International Journal of Clinical and Experimental Hypnosis. 2020;68:419–32.					X
Trevino C, Geier T, Timmer-Murillo SC, Shawlin M, Milia DJ, Codner P, et al. Feasibility of a trauma quality-of-life follow-up clinic. J Trauma Acute Care Surg. 2020;89:226–9.			X		
Turpin G, Downs M, Mason S. Effectiveness of providing self-help information following acute traumatic injury: Randomised controlled trial. Br J Psychiatry. 2005;187:76–82.					X
Villain M, Sibon I, Renou P, Poli M, Swendsen J. Very early social support following mild stroke is associated with emotional and behavioral outcomes three months later. Clin Rehabil. 2017;31:135–41.				X	
Visser E, Gosens T, Den Oudsten B, De Vries J. Physical Trauma Patients with Symptoms of an Acute and Posttraumatic Stress Disorder: Protocol for an Observational Prospective Cohort Study. JMIR Res Protoc. 2018;7:e88.		X			
Wagner AW, Zatzick DF, Ghesquiere A, Jurkovich GJ. Behavioral Activation as an Early Intervention for Posttraumatic Stress Disorder and Depression Among Physically Injured Trauma Survivors. Cognitive and Behavioral Practice. 2007;14:341–9.					X
Wain HJ, Gabriel GM. Psychodynamic Concepts Inherent in a Biopsychosocial Model of Care of Traumatic Injuries. The Journal of the American Academy of Psychoanalysis and Dynamic Psychiatry. 2007;35:555–73.					X
Wain H J, Grammer GG, Stasinos J, DeBoer CM. Psychiatric Intervention for medical and surgical Patients following traumatic Injuries. In: Interventions following mass violence and Deisasters: Strategies for mental health practice. 2006.					X
Wake E, Battistella T, Dale K, Scott M, Nelson R, Marshall AP. Evaluation of a Trauma Service: Patient and Family Perspectives. J Trauma Nurs. 2020;27:216–24.	X				
Walker K, Stephenson M, Loupis A, Ben-Meir M, Joe K, Stephenson M, et al. Displaying emergency patient estimated wait times: A multi-centre, qualitative study of patient, community, paramedic and health administrator perspectives. Emerg Med Australas. 2020. https://doi.org/10.1111/1742-6723.13640.				X	
Wegener ST, Pollak AN, Frey KP, Hymes RA, Archer KR, Jones CB, et al. The Trauma Collaborative Care Study (TCCS). J Orthop Trauma. 2017;31 Suppl 1:S78–87.				X	
Weinerman J, Vazquez A, Schurhoff N, Shatz C, Goldenberg B, Constantinescu D, et al. The impacts of anxiety and depression on outcomes in orthopaedic trauma surgery: a narrative review. Ann Med Surg (Lond). 2023;85:5523–7.				X	

Reference	Reason of exclusion				
	<18	Nft	R1	R2	R3
Weisz GM, Boyd DR. Psyche-trauma-psyche: surgeons’ observations of psychiatric conditions in trauma patients. Isr Ann Psychiatr Relat Discip. 1973;11:91–8.				X	
Wijesinghe CA, Williams SS, Kasturiratne A, Dolawaththa N, Wimalaratne P, Wijewickrema B, et al. A Randomized Controlled Trial of a Brief Intervention for Delayed Psychological Effects in Snakebite Victims. PLoS Negl Trop Dis. 2015;9:e0003989.					X
Wilczkiewicz E. Emotion Expression in Post-MI/PCI patients – the Effects of Two Types of Social Sharing on Psychological and Physiological Outcomes.					X
Wiman E, Wikblad K, Idvall E. Trauma patients’ encounters with the team in the emergency department—A qualitative study. International Journal of Nursing Studies. 2007;44:714–22.				X	
Winje D. Cognitive coping: The psychological significance of knowing what happened in the traumatic event. J Traum Stress. 1998;11:627–43.	X				
Wisely JA, Tarrier N. A survey of the need for psychological input in a follow-up service for adult burn-injured patients. Burns. 2001;27:801–7.	X				
Wisely JA, Hoyle E, Tarrier N, Edwards J. Where to start? Attempting to meet the psychological needs of burned patients. Burns. 2007;33:736–46.				X	
Wong EM-L, Chan SW-C, Chair S-Y. Effectiveness of an educational intervention on levels of pain, anxiety and self-efficacy for patients with musculoskeletal trauma. Journal of Advanced Nursing. 2010;66:1120–31.					X
Worsham CM, Banzett RB, Schwartzstein RM. Dyspnea, Acute Respiratory Failure, Psychological Trauma, and Post-ICU Mental Health: A Caution and a Call for Research. Chest. 2021;159:749–56.				X	
Yang Y, Tang T-T, Chen M-R, Xiang M-Y, Li L-L, Hou X-L. Prevalence and association of anxiety and depression among orthopaedic trauma inpatients: a retrospective analysis of 1994 cases. J Orthop Surg Res. 2020;15:587.	X				
Zanza C, Romenskaya T, Zuliani M, Piccolella F, Bottinelli M, Caputo G, et al. Acute Traumatic Pain in the Emergency Department. Diseases. 2023;11:45.				X	
Zatzick D, Jurkovich G, Heagerty P, Russo J, Darnell D, Parker L, et al. Stepped Collaborative Care Targeting Posttraumatic Stress Disorder Symptoms and Comorbidity for US Trauma Care Systems: A Randomized Clinical Trial. JAMA Surg. 2021;156:430.					X
Zatzick D, Jurkovich G, Rivara FP, Russo J, Wagner A, Wang J, et al. A Randomized Stepped Care Intervention Trial Targeting Posttraumatic Stress Disorder for Surgically Hospitalized Injury Survivors. Annals of Surgery. 2013;257:390–9.					X

Reference	Reason of exclusion				
	<18	Nft	R1	R2	R3
Zatzick D, Rivara F, Jurkovich G, Russo J, Trusz SG, Wang J, et al. Enhancing the population impact of collaborative care interventions: mixed method development and implementation of stepped care targeting posttraumatic stress disorder and related comorbidities after acute trauma. General Hospital Psychiatry. 2011;33:123–34.			X		
Zatzick D, Roy-Byrne P, Russo J, Rivara F, Droesch R, Wagner A, et al. A Randomized Effectiveness Trial of Stepped Collaborative Care forAcutely Injured Trauma Survivors. Arch Gen Psychiatry. 2004;61:498.					X
Zdziarski-Horodyski L, Horodyski M, Sadasivan KK, Hagen J, Vasilopoulos T, Patrick M, et al. An integrated-delivery-of-care approach to improve patient reported physical function and mental wellbeing after orthopedic trauma: study protocol for a randomized controlled trial. Trials. 2018;19:32.					X
Zhang L, Zhou J. Crisis Intervention in the Acute Stage after Trauma. Int J Emerg Ment Health. 2015;17.					X
Zimmermann CJ, Zelenski AB, Buffington A, Baggett ND, Tucholka JL, Weis HB, et al. Best case/worst case for the trauma ICU: Development and pilot testing of a communication tool for older adults with traumatic injury. J Trauma Acute Care Surg. 2021;91:542–51.			X		

<18: Article includes patients under the age of 18; Nft: No full text available/Protocol; R1: No psychosocial outcome; R2: No recommendation for a psychosocial intervention or communication methodology; R3: No emergency patient/acute setting
