# Supplementary material for: Psychological care in acute and emergency medicine: a scoping review of support interventions by healthcare professionals
Source: BMC Emerg Med. 2026 Feb 18;26:64. doi: 10.1186/s12873-026-01494-y (PMC12930831; doi:10.1186/s12873-026-01494-y)
Supplement: Supplementary file 2 — Supplementary Material 2 [file 12873_2026_1494_MOESM2_ESM.pdf]

Supplementary material 2: Characteristics of included articles

Table 1

Trauma department

Population	Intervention	Main Outcome / Key message	Number of patients	Providers	Survey period [y] / Country	Study design
**Braaf et al. 2018 Patient-identified information and communication needs in the context of major trauma**						
Major trauma survivors	Multimodal communication, consistent information provision and sharing, active communication, written discharge plans, presence of specialist trauma coordinator or trauma patient advocate	Recommendations to prevent distress and insecurity: Written discharge plans, presence of specialist trauma coordinator or trauma patient advocate, use of multi-modal communication strategies, plain language and consistency of information.	65	Clinical healthcare professionals	2014-2015 Australia	Longitudinal qualitative with phenomenological approach
**Yadav & Shrestha 2017 A study on posttraumatic experience of road traffic accident afflicted maxillofacial trauma patient at tertiary hospital**						
Oral and maxillofacial trauma patients following road traffic accident	Explaining and informing about missing memoirs, repeated preoperative education, building a trustworthy personnel-patient interaction	Patients want healthcare professionals to provide them with information and assist them in informing their family members, colleagues or friends to make personal arrangements. Preoperative education did not reduce anxiety.	20	Clinical healthcare professionals	2015 Nepal	Qualitative phenomenological approach
**Vincent et al. 2015 Psychological Distress After Orthopedic Trauma: Prevalence in Patients and Implications for Rehabilitation**						
Orthopaedic trauma patients	Counselling, pastoral care, coping skills for pain, self-management, group support and networks, meditation and mindfulness, education and information about trauma and recovery	Counselling twice a week for 45 minutes reduces anxiety and depression. Pastoral care has positive effects on PTSD symptoms. Education about trauma and recovery helps patients understand their symptoms. Meditation helps patients overcome fears and emotions blocking recovery, while mindfulness enhances present focus and bodily awareness.	n. a.	Pastor, clinical psychologists, healthcare professionals	n. a. USA	Narrative review
**deRoon-Cassini et al. 2019 Screening and treating hospitalized trauma survivors for posttraumatic stress disorder and depression**						
Patients with traumatic injury	Modified Prolonged Exposure (PE), Behavioural Activation (BA), stepped collaborative care, multitier approach to psychological intervention after traumatic-injury (MAPIT), Screening	Modified PE is effective at reducing PTSD symptom severity by one month. BA improved mood and physical functioning. Stepped collaborative care reduces PTSD symptoms and improves functional improvements. MAPIT	n. a.	Psychologists, social workers, nurses, psychiatrists	n. a. USA	Narrative review

Population	Intervention	Main Outcome / Key message	Number of patients	Providers	Survey period [y] / Country	Study design
		needs further research. Hospital screening is most effective initially.				
**Manser et al. 2018 Do screening and a randomized brief intervention at a Level 1 trauma center impact acute stress reactions to prevent later development of post-traumatic stress disorder?**						
Trauma patients admitted to the hospital with at least one symptom of acute stress based on screening with the PC-PTSD	60-minute bedside consulting focusing on engagement, symptom education and normalization, emotional safety coping strategies, and an individualized referral, if desired, to a community mental health provider aiming to reduce initial distress and promote adaptive coping in the acute aftermath of trauma	The brief intervention did not reduce or prevent the development of PTSD.<br>The intervention group had a significantly greater improvement in PTSD symptom scores from the 45- to 90-day interview compared to the control group, even though the reduction in symptoms was not clinically relevant.	IG (80)<br>CG (60)	Clinical coordinators and social work graduate research assistants	2015-2017<br>USA	Prospective RCT
**Tecic et al. 2011 Early Short-Term Inpatient Psychotherapeutic Treatment Versus Continued Outpatient Psychotherapy on Psychosocial Outcome: A Randomized Controlled Trial in Trauma Patients**						
Trauma patients with minimum two injuries with a combined Abbreviated Injury Scale (AIS) Severity Score Index ≥5	Inpatient psychotherapy: supportive and stabilizing elements, counselling, cognitive reorganization, imagination, resource activation, exposure, and relaxation techniques	Anxiety symptoms decreased significantly for all patients. Patients in the long-term psychotherapy group had a better outcome regarding anxiety, depression and PTSD than short-term patients. Implementation of psychotherapy session in surgical ward is not recommended due to interruptions and disturbances. The intervention should be gender-specific and flexible.	Short-term (59)<br>Long-term (54)	Psychotherapist trained in psychotraumtology	2001-2005<br>Germany	RCT
**Frank et al. 2017 Addressing Traumatic Stress in the Acute Traumatically Injured Patient**						
Trauma patients<br>51% motor vehicle crash<br>21% falls	Primary Care-PTSD (PC-PTSD) Screening followed by a health psychology consult if risk for PTSD is confirmed.	Implementing the PC-PTSD tool increases nurses' awareness of patients' psychological health, leading to better referrals and improved outcomes for patients. Providing holistic care for trauma patients helps them cope and return to a high quality of life.	39	Nurses (bedside)	2016<br>USA	Pre-/Post descriptive approach
**Tanti et al. 2023 Understanding Patients' Perspectives of Clinical Communication within a Major Trauma Centre**						
Major trauma patients	Written information<br>Journal<br>Clear explanation of injuries using different modalities	Patients desire information to be provided in various modes and in a clear language. They request healthcare professionals to reassure patients, utilize humour, show humanity and to	20	Clinical healthcare professionals	2022-2023<br>United Kingdom	Qualitative

Population	Intervention	Main Outcome / Key message	Number of patients	Providers	Survey period [y] / Country	Study design
	Two-way person centred conversations	communicate in a caring and helpful ways. They should respond to vulnerability, powerlessness, and dependency with empathy, kindness, and respect. Patients should be encouraged to engage in their treatment.				
**Obey & Miller 2022 Resources for Patient Mental Health and Well-being after Orthopaedic Trauma**						
Orthopaedic trauma patients	Counselling and chaplain visits<br>Client-centered-therapy<br>Trauma Collaborative Care program (TCC)<br>Trauma Recovery Services (TRS) program<br>Trauma-informed care (TIC)<br>Meditation and mindfulness<br>Trauma Survivors Network (TSN): peer visitation and family support	Pastoral care had positive effects on patients' symptoms and personal growth. Counselling/client-centered therapy had a positive impact on the quality of life. Anxiety depression and reported pain levels were decreased. TCC showed a small positive effect on early outcomes of utilization of TSN resources. TRS can increase patient-reported ratings of care and satisfaction after hospital discharge. Meditation can reduce symptoms of depression and PTSD. TSN reduces the likelihood of depression by 49%.	n. a.	Orthopaedic surgeons and other members of the care team	n. a.<br>USA	Narrative review
**Timmer-Murillo et al. 2023 Comprehensive Framework of Firearm Violence Survivor Care. A Review**						
Firearm violence survivors	Trauma-informed care (TIC): modify communication to improve rapport and comfort, time for questions, involving patients in discussions on care, informing about procedures; screening of risk of psychopathology and reinjury; psychological intervention: multitier approach to psychological intervention model, stepped-care approach (screening, features of CBT and motivational interviewing strategies)	The comprehensive care interventions show improvement in patients' long-term recovery. More patients have access to the psychological care they need. It acts as a means of tertiary prevention of firearm violence.	n. a.	Healthcare professionals	n. a.<br>USA	Narrative review

COPD: chronic obstructive pulmonary disease; ICU: Intensive care unit; IG: intervention group; CG: control group, n. a.: Not applicable; NIV: non-invasive-ventilation; n. r.: Not reported, RCT: Randomized controlled trial, PTSD: Posttraumatic stress disorder, CBT: Cognitive behavioural therapy, EMDR: Eye movement desensitization and reprocessing, PCLS: Post concussion-like symptoms, MAPIT: Multitier approach to psychological intervention after traumatic-injury

Table 2

Burn unit

Popu-lation	Intervention	Main Outcome/ Key message	Number of pa-tients	Providers	Survey period [y] / Country	Study design
**Gullick et al. 2014 The trauma bubble: patient and family experience of serious burn injury**						
Burn pa-tients	Available specialist psychologist with a proactive management strategy. Thera-peutic approach for patients with facial burns. Burn patients and families should be engaged in discussions about possible emotional trauma, trough written re-sources.	It is recommended that patients receive psychological first aid, which should include physical and emotional comfort, perceived safety, and early information about possible emotional reactions to trauma. Encouraging contact with family and friends should be provided for patients. There should be a proactive offer from a specialist psychologist.	9 pa-tients, 9 carers	Psycholo-gists, Burn clinicians	n. r. Australia	Qualita-tive, phe-nomeno-logical
**Cleary et al. 2020 Before, during and after: Trauma-informed care in burns settings**						
Burn pa-tients	TIC (trauma-informed care)<br>1. Before contact to patient: clinical inte-gration of TIC principles<br>2. During: Creating safety, screening, psychoeducation and collaboration with patient and supporters, use of peer-led services, minimise the impact of trauma<br>3. After: Proactive support trough out-reach and follow-up	Advised in the process of interaction and intervention with the patient: creating safety; screening; collaborative inclusion of the patient in the intervention and recovery planning process; psy-choeducation of the patient and supporters; and the use of peer-led services. TIC has the potential to minimise psychological distress and the harms associated with burn injuries, and to pro-actively support patient recovery in the short and long term.	n. a.	Healthcare professionals	n. a. Australia	Narrative review
**Johnson et al. 2016 Emerging from the trauma bubble: Redefining 'normal' after burn injury**						
Burn pa-tients	Early psychological support including psychological first aid, early discussions about emotional trauma, provision of written resources	Early psychological support is advised. Healthcare professionals should engage family in being close to the patient and patients in early self-care. Provision of written resources can provide a use-ful precursor to psychological referral.	9 pa-tients, 9 close family members	Psycholo-gists, burn clinicians	n. r. Australia	Qualita-tive

COPD: chronic obstructive pulmonary disease; ICU: Intensive care unit; IG: intervention group; CG: control group, n. a.: Not applicable; NIV: non-invasive-ventilation; n. r.: Not reported, RCT: Randomized controlled trial, PTSD: Posttraumatic stress disorder, CBT: Cognitive behavioural therapy, EMDR: Eye movement desensitization and repro-cessing, PCLS: Post concussion-like symptoms, MAPIT: Multitier approach to psychological intervention after traumatic-injury

Table 3

Emergency medical service

Population	Intervention	Main Outcome/ Key message	Number of patients	Provider	Survey period [y] / Country	Study design
**Visser et al. 2021 Patients' experiences and wellbeing after injury: A focus group study**						
Shook room patients	Informing patients about injury and treatment	Patients want more information and communication about injury and treatment in ambulance and shock room, especially if they have memory loss.	28	Physicians and nurses	n. r. Netherlands	Focus group
**Arimon et al. 2021 A Communicative Intervention to Improve the Psychoemotional State of Critical Care Patients Transported by Ambulance**						
Critical care patients transported by ambulance	CONECTEM communicative intervention: augmentative alternative communication and basic communication skills adapted to the Glasgow Coma Scale.	CONECTEM Communication intervention led to a significant decrease in anxiety and PTSD symptoms.	IG (68) CG (52)	Emergency medical team nurses	n. r. Spain	Quasi-experimental
**Nutbeam et al. 2022 Understanding people's experiences of extrication while being trapped in motor vehicles: a qualitative interview study**						
Patients who had been trapped in a vehicle following an motor vehicle crash	Positive communication and reassurance during the extraction of patients from a motor vehicle	Positive communication, reassurance and a planned companionship for patients are important aspects of psychosocial care while extricating. Patients should be informed about the safety of co-occupants, allowed to communicate with family if conscious, protected from public photography, and offered follow-up, with rescuers refraining from posting extrication related photos on social media.	8	Emergency workers, bystanders	2020 – 2021 United Kingdom	Qualitative

COPD: chronic obstructive pulmonary disease; ICU: Intensive care unit; IG: intervention group; CG: control group, n. a.: Not applicable; NIV: non-invasive-ventilation; n. r.: Not reported, RCT: Randomized controlled trial, PTSD: Posttraumatic stress disorder, CBT: Cognitive behavioural therapy, EMDR: Eye movement desensitization and reprocessing, PCLS: Post concussion-like symptoms, MAPIT: Multitier approach to psychological intervention after traumatic-injury

Table 4

Emergency department

Population	Intervention	Main Outcome/ Key message	Number of patients	Provider	Survey period [y] / Country	Study design
**Visser et al. 2017 The course, prediction, and treatment of acute and posttraumatic stress in trauma patients: A systematic review**						
Trauma patients	Psychoeducation, CBT, supportive counselling, hypnosis, psychoeducation in combination with psychotherapy, EMDR	Recommendation of psychoeducation through nurses, secondly referring for further treatment if needed. Early treatment, within the first two weeks after the trauma, can prevent PTSD. A feasible stepped care intervention or CBT elements such as psychoeducation could overcome barriers. Effective treatment: psychoeducation alone and in combination with psychotherapy, CBT alone and in combination with hypnosis, EMDR. Ineffective treatment: self-help booklets and internet CBT.	n. a.	Nurses	1993 – 2015 Netherlands	Systematic review
**Gil-Jardiné et al. 2018 Emergency room intervention to prevent post concussion-like symptoms and post-traumatic stress disorder. A pilot randomized controlled study of a brief eye movement desensitization and reprocessing intervention versus reassurance or usual care**						
Patients with a high risk of PCLS	Reassurance session (psychoeducation and cognitive distortions) and EMDR	A single session of EMDR R-TEP psychotherapy performed at the ED reduces the rate of PTSD and PCLS symptoms.	109 randomized into R-TEP EMDR (34), reassurance (38), treatment as usual (37)	Emergency room professionals (therapist and psychologists)	2016 France	RCT
**Figueroa et al. 2022 The ABCDE psychological first aid intervention decreases early PTSD symptoms but does not prevent it: results of a randomized-controlled trial**						
Participants who were exposed to a recent event (≤ 72 hours) that imposed an actual or threatened death or serious injury directly, as a witness, or as a close relative /friend who suddenly learned about a mishap that affected a loved one	PFA-ABCDE:<br>A: Active Listening<br>B: Breathing retraining<br>C: Categorization of needs<br>D: *Derivación* (referral)<br>E: (psycho-) education	Higher immediate distress relief, fewer PTSD symptoms at one-month follow-up but no significant difference of depressive symptoms at one-month follow-up or PTSD symptoms at six-month follow-up.	221<br>IG (109)<br>CG (psychoeducation) (112)	Psychology students with training in PFA-ABCDE	n. r. Chile	RCT

Population	Intervention	Main Outcome/ Key message	Number of patients	Provider	Survey period [y] / Country	Study design
**Willinge et al. 2024 Orthopaedic trauma patients' experiences with emergency department care and follow-up through Virtual Fracture Care review: a qualitative study**						
Orthopaedic trauma patients	Humour, plain language, sharing medical images, a primarily responsible health professional, clarity of diagnosis	Recommendations: 1. Anticipating evolving information needs, 2. Engaging patients early to clarify care processes, 3. Involving patients in treatment and decision-making, and 4. broadening information and scheduling	15	Emergency department health professionals	2022 Netherlands	Qualitative

COPD: chronic obstructive pulmonary disease; ICU: Intensive care unit; IG: intervention group; CG: control group, n. a.: Not applicable; NIV: non-invasive-ventilation; n. r.: Not reported, RCT: Randomized controlled trial, PTSD: Posttraumatic stress disorder, CBT: Cognitive behavioural therapy, EMDR: Eye movement desensitization and reprocessing, PCLS: Post concussion-like symptoms, MAPIT: Multitier approach to psychological intervention after traumatic-injury

Table 5

Intensive care unit

Population	Intervention	Main outcome/ key message	Number of patients [n] or IG[n]/CG[n]	Providers	Survey period [y]	Study design
**Peris et al. 2011 Early intra-intensive care unit psychological intervention promotes recovery from posttraumatic stress disorders, anxiety and depression symptoms in critically ill patients**						
Major trauma patients with need for mechanical ventilation	Educational interventions, counselling, stress management, psychological support, coping strategies	Early intra-ICU psychological intervention can decrease the risk of PTSD, anxiety and depression at 12 months after ICU discharge. An intra-ICU clinical psychologist may help patients to recover from acute, stressful experiences.	IG (123)<br>CG (86)	Nurses<br>Clinical psychologists	2005-2009<br>Italy	Observational study
**Mohta et al. 2003 Psychological care in trauma patients**						
Trauma patients	Informing patients, communication with respect, psychological interventions<br>In case of amputation: counselling and psychotherapeutic interventions	Knowing what is happening reduces the feeling of anxiety, helplessness and sense of immobilisation. Family visits should be allowed as soon as possible. The usual day-night-cycle should be maintained whenever possible. To support the dignity of patients, healthcare professionals should communicate their respect for the patients, e.g. through eye contact and sitting down instead of standing while feeding.	n. a.	ICU healthcare professionals	n. a.<br>India	Narrative review

Population	Intervention	Main outcome/ key message	Number of patients [n] or IG[n]/CG[n]	Providers	Survey period [y]	Study design
**Cekic et al. 2022 The Effect of Provision of Information and Supportive Nursing Care on Blood Gas, Vital Signs, Anxiety, Stress, and Agitation Levels in COPD Patients Treated with NIV: A Randomized Controlled Trial**						
COPD patients treated for the first time with NIV	Additional information, supportive nursing care	Providing information and supportive nursing care reduced anxiety, stress, and agitation in COPD patients treated with NIV.	IG (30)<br>CG (30)	Researchers	2019<br>Turkey	RCT

COPD: chronic obstructive pulmonary disease; ICU: Intensive care unit; IG: intervention group; CG: control group, n. a.: Not applicable; NIV: non-invasive-ventilation; n. r.: Not reported, RCT: Randomized controlled trial, PTSD: Posttraumatic stress disorder, CBT: Cognitive behavioural therapy, EMDR: Eye movement desensitization and reprocessing, PCLS: Post concussion-like symptoms, MAPIT: Multitier approach to psychological intervention after traumatic-injury

Table 6

Other departments with acute or emergency medicine context

Setting	Population	Intervention	Main Outcome/ Key message	Number of patients	Provider	Survey period [y] / Country	Study design
**Wade et al. 2013 Early response to psychological trauma** What GPs can do							
General practitioner	Primary care patients following a traumatic event	Stepped care approach:<br>1. Early response: advice and support<br>2. Simple psychological strategies<br>3. Formal mental health interventions	General practitioners can assist in providing a sense of safety and self-efficacy, teaching calming strategies, promoting hope, helping to connect patients to social support, and monitoring patients who have experienced a traumatic situation. Offering psychological debriefing on a routine basis during the initial weeks following a traumatic event is not advised.	n. a.	General practitioners	n. a.<br>Australia	Narrative review

Setting	Population	Intervention	Main Outcome/ Key message	Number of patients	Provider	Survey period [y] / Country	Study design
**Gamble et al. 2005 Effectiveness of a Counseling Intervention after a Traumatic Childbirth: A Randomized Controlled Trial**							
Postnatal ward	Women during their last trimester of pregnancy	Counselling within 72 hours of birth: critical stress debriefing and issues relevant to the childbearing context	Counselling intervention by midwifes within 72 hours of birth was effective in reducing symptoms of trauma, depression, stress and feelings of self-blame.	IG (50) CG (53)	Midwifes	2001-2002 Australia	RCT
**Gartlehner et al. 2013 Interventions for the Prevention of PTSD in Adults after exposure to psychological trauma**							
Outpatient and inpatient primary care specialty mental health care settings, community settings (e.g., churches, community health centres, rape crisis centres), military settings	Adults exposed to psychological trauma (interpersonal or domestic violence or abuse; sexual abuse or assault; rape; combat- or military-related trauma; crime-related events; terrorism; slavery; natural disasters; injury; life-threatening illness; captivity; life-threatening medical procedures; witnessing a traumatic event; refugee trauma; prisoner of war-related trauma; asylum seeking-related trauma)	Debriefing<br>Brief trauma-focused CBT<br>Supportive counselling<br>Collaborative care	Brief trauma-focused CBT might be a preferable choice to reduce PTSD symptoms. Debriefing is not an effective prevention intervention. Collaborative care might be preferred for trauma patients requiring surgical hospitalization	n. a.	Healthcare professionals	n. a. USA	Systematic review & Meta-analysis

COPD: chronic obstructive pulmonary disease; ICU: Intensive care unit; IG: intervention group; CG: control group, n. a.: Not applicable; NIV: non-invasive-ventilation; n. r.: Not reported, RCT: Randomized controlled trial, PTSD: Posttraumatic stress disorder, CBT: Cognitive behavioural therapy, EMDR: Eye movement desensitization and reprocessing, PCLS: Post concussion-like symptoms, MAPIT: Multitier approach to psychological intervention after traumatic-injury
