# Supplementary material for: Psychological care in acute and emergency medicine: a scoping review of support interventions by healthcare professionals
Source: BMC Emerg Med. 2026 Feb 18;26:64. doi: 10.1186/s12873-026-01494-y (PMC12930831; doi:10.1186/s12873-026-01494-y)
Supplement: Supplementary file 4 — Supplementary Material 4 [file 12873_2026_1494_MOESM4_ESM.docx]

**Supplement material 5: Glossary about the Emergency Settings included**

| Emergency setting | Definition |
| --- | --- |
| Trauma department | A trauma department delivers rapid assessment and definitive care for patients with severe blunt or penetrating injuries. It provides advanced diagnostics, surgical and critical care resources, and multidisciplinary expertise to stabilize compromised physiology and perform timely interventions, including fracture management [91, 92]. |
| Burn department | Burn units are equipped with a specialized set of resources, including expertise in surgical and nursing wound care, critical care, and advanced trauma rehabilitation. While developed primarily for burn patients, these capabilities are also applicable to a wide range of non-burn medical and surgical conditions [93]. |
| Emergency department | The emergency department is a core hospital unit that provides continuous, 24-hour care for patients presenting with unexpected illnesses and injuries, serving as a critical link between primary and specialist care [94]. |
| Intensive care unit | An intensive care unit (ICU) is a structured care system for critically ill patients, providing specialized medical and nursing interventions, advanced monitoring, and multiple forms of organ support to sustain life during severe organ dysfunction. Although typically located within a defined hospital area, ICU care often extends beyond this space to include the emergency department, hospital wards, and follow-up services [95]. |
| Emergency medical service | Emergency medical services comprise an organized system responsible for responding to emergency calls and conducting patient transports, including both life-threatening and urgent cases [96]. Their primary aim is to deliver rapid intervention in life-threatening and urgent situations to prevent unnecessary mortality and long-term morbidity [97]. |
| Other care settings | Settings that provide care for medical emergencies but do not fit into any of the previously defined categories, or that span multiple categories. |

**References**

91. Raschke MJ, Haas NP. Unfallchirurgie. In: Siewert JR, Stein HJ, editors. Chirurgie. Berlin, Heidelberg: Springer Berlin Heidelberg; 2012. p. 865–999. https://doi.org/10.1007/978-3-642-11331-4_8.

92. Morgan JM, Calleja P. Emergency trauma care in rural and remote settings: Challenges and patient outcomes. Int Emerg Nurs. 2020;51:100880. https://doi.org/10.1016/j.ienj.2020.100880.

93. Sheridan RL, Greenhalgh D. Special Problems in Burns. Surg Clin North Am. 2014;94:781–91. https://doi.org/10.1016/j.suc.2014.05.002.

94. Kefyalew M, Gidey U, Azazh A, Kaki M, Mesfin N. Reducing the length of stay of cardiac patients in the Adult Emergency department by using a Quality improvement framework. Int Emerg Nurs. 2023;71:101368. https://doi.org/10.1016/j.ienj.2023.101368.

95. Marshall JC, Bosco L, Adhikari NK, Connolly B, Diaz JV, Dorman T, et al. What is an intensive care unit? A report of the task force of the World Federation of Societies of Intensive and Critical Care Medicine. J Crit Care. 2017;37:270–6. https://doi.org/10.1016/j.jcrc.2016.07.015.

96. Reuter-Oppermann M, Van Den Berg PL, Vile JL. Logistics for Emergency Medical Service systems. Health Syst. 2017;6:187–208. https://doi.org/10.1057/s41306-017-0023-x.

97. Al-Shaqsi S. Models of International Emergency Medical Service (EMS) Systems. Oman Med J. 2010. https://doi.org/10.5001/omj.2010.92.
